# Supplementary material for: Gender-transformative health promotion interventions for linking and retaining tuberculosis-diagnosed adult men in care in sub-Saharan Africa: A scoping review protocol
Source: PLoS One. 2026 Jan 8;21(1):e0339666. doi: 10.1371/journal.pone.0339666 (PMC12782366; doi:10.1371/journal.pone.0339666)
Supplement: S1 Fig — (PDF) [file pone.0339666.s001.pdf]

UNIVERSITY OF KWAZULU-NATAL ™
INYUVESI YAKWAZULU-NATALI

24 June 2025

Mr Siyabonga Kave (215082460)
School of Nursing & Public Health
Howard College

Dear Mr Kave,

Protocol reference number: BREC/00008572/2025
Project title: Developing a gender-transformative health promotion intervention to retain tuberculosis diagnosed men in care in Eastern Cape, South Africa
Degree: PhD

EXPEDITED APPLICATION

A sub-committee of the Biomedical Research Ethics Committee has considered and noted your application.

The conditions have been met and the study is given full ethics approval and may begin as from 24 June 2025. Please ensure that any outstanding site permissions are obtained and forwarded to BREC for approval before commencing research at a site.

This approval is valid for one year from 24 June 2025. To ensure uninterrupted approval of this study beyond the approval expiry date, an application for recertification must be submitted to BREC on RIG on the appropriate BREC form 2-3 months before the expiry date.

Any amendments to this study, unless urgently required to ensure safety of participants, must be approved by BREC prior to implementation.

Your acceptance of this approval denotes your compliance with South African National Research Ethics Guidelines (2024), South African National Good Clinical Practice Guidelines (2020) (if applicable) and with UKZN BREC ethics requirements as contained in the UKZN BREC Terms of Reference and Standard Operating Procedures, all available at https://research.ukzn.ac.za/research-office/ethics-overview/biomedical-research-ethics/.

BREC is registered with the South African National Health Research Ethics Council (REC-290408-009). BREC has US Office for Human Research Protections (OHRP) Federal-wide Assurance (FWA 678).

The sub-committee's decision will be noted by a full Committee at its next meeting taking place on 08 July 2025.

Yours sincerely,

Prof S Singh
Chair: Biomedical Research Ethics Committee

Biomedical Research Ethics Committee
Chair: Professor S Singh
UKZN Research Ethics Office Westville Campus, Govan Mbeki Building
Postal Address: Private Bag X54001, Durban 4000
Email: BREC@ukzn.ac.za
Website: https://research.ukzn.ac.za/research-office/ethics-overview/biomedical-research-ethics/
