# Supplementary material for: Gender-transformative health promotion interventions for linking and retaining tuberculosis-diagnosed adult men in care in sub-Saharan Africa: A scoping review protocol
Source: PLoS One. 2026 Jan 8;21(1):e0339666. doi: 10.1371/journal.pone.0339666 (PMC12782366; doi:10.1371/journal.pone.0339666)
Supplement: S2 Fig — (DOCX) [file pone.0339666.s002.docx]

**PRISMA-P (Preferred Reporting Items for Systematic review and Meta-Analysis Protocols) 2015 checklist: recommended items to address in a systematic review protocol***

| Section and topic | Item No | Checklist item | Completed items by the author |
| --- | --- | --- | --- |
| ADMINISTRATIVE INFORMATION | | | |
| Title: |  |  |  |
| Identification | 1a | Identify the report as a protocol of a systematic review | Protocol for a scoping review on gender-transformative TB care for men in SSA |
| Update | 1b | If the protocol is for an update of a previous systematic review, identify as such | Not applicable – this is a new protocol |
| Registration | 2 | If registered, provide the name of the registry (such as PROSPERO) and registration number | Completed: Registered with Open Science Framework, DOI: https://doi.org/10.17605/OSF.IO/RNF2T |
| Authors: |  |  |  |
| Contact | 3a | Provide name, institutional affiliation, e-mail address of all protocol authors; provide physical mailing address of corresponding author | Completed:  Siyabonga Kave,  e-mail: siyakave@gmail.com,  Cellphone: +27839849921.   1. School of Nursing and Public Health, College of Health Sciences, Howard College Campus, University of KwaZulu-Natal, Durban, South Africa.   Yandisa Sikweyiya,   1. School of Nursing and Public Health, College of Health Sciences, Howard College Campus, University of KwaZulu-Natal, Durban, South Africa 2. Gender and Health Research Unit, South African Medical Research Council, Pretoria, South Africa 3. School of Public Health, University of the Witwatersrand, Johannesburg, South Africa   Nelisiwe Khuzwayo;   1. School of Nursing and Public Health, College of Health Sciences, Howard College Campus, University of KwaZulu-Natal, Durban, South Africa |
| Contributions | 3b | Describe contributions of protocol authors and identify the guarantor of the review | Completed: SK, YS, and NK conceptualized and designed the study; all contributed to manuscript development |
| Amendments | 4 | If the protocol represents an amendment of a previously completed or published protocol, identify as such and list changes; otherwise, state plan for documenting important protocol amendments | Not applicable – this is a new protocol |
| Support: |  |  |  |
| Sources | 5a | Indicate sources of financial or other support for the review | Not applicable |
| Sponsor | 5b | Provide name for the review funder and/or sponsor | Not applicable |
| Role of sponsor or funder | 5c | Describe roles of funder(s), sponsor(s), and/or institution(s), if any, in developing the protocol | Not applicable |
| INTRODUCTION | | |  |
| Rationale | 6 | Describe the rationale for the review in the context of what is already known | Gender norms and masculine identities impact men’s TB care in SSA; there is a need for a synthesis of gender-transformative interventions. |
| Objectives | 7 | Provide an explicit statement of the question(s) the review will address with reference to participants, interventions, comparators, and outcomes (PICO) | What evidence exists on gender-transformative and health promotion interventions for linking/retaining men with TB in SSA? |
| METHODS | | |  |
| Eligibility criteria | 8 | Specify the study characteristics (such as PICO, study design, setting, time frame) and report characteristics (such as years considered, language, publication status) to be used as criteria for eligibility for the review | Studies on men (18 years and above) diagnosed with TB; conducted in SSA; published 2015–2024; English-language |
| Information sources | 9 | Describe all intended information sources (such as electronic databases, contact with study authors, trial registers or other grey literature sources) with planned dates of coverage | PubMed, MEDLINE, Web of Science, PsychInfo, Google Scholar, WHO databases |
| Search strategy | 10 | Present draft of search strategy to be used for at least one electronic database, including planned limits, such that it could be repeated | Provided in Annexure S1 (search strategy) |
| Study records: |  |  |  |
| Data management | 11a | Describe the mechanism(s) that will be used to manage records and data throughout the review | EndNote for organizing studies; Excel for data extraction |
| Selection process | 11b | State the process that will be used for selecting studies (such as two independent reviewers) through each phase of the review (that is, screening, eligibility and inclusion in meta-analysis) | Three reviewers independently screen and resolve conflicts through consensus |
| Data collection process | 11c | Describe planned method of extracting data from reports (such as piloting forms, done independently, in duplicate), any processes for obtaining and confirming data from investigators | Standardized charting form used by three reviewers |
| Data items | 12 | List and define all variables for which data will be sought (such as PICO items, funding sources), any pre-planned data assumptions and simplifications | Author, year, population, intervention type, setting, design, outcomes |
| Outcomes and prioritization | 13 | List and define all outcomes for which data will be sought, including prioritization of main and additional outcomes, with rationale | Types and effectiveness of gender-transformative and health promotion interventions |
| Risk of bias in individual studies | 14 | Describe anticipated methods for assessing risk of bias of individual studies, including whether this will be done at the outcome or study level, or both; state how this information will be used in data synthesis | MMAT (Mixed Methods Appraisal Tool) will be used |
| Data synthesis | 15a | Describe criteria under which study data will be quantitatively synthesised | Not applicable : Narrative synthesis planned |
|  | 15b | If data are appropriate for quantitative synthesis, describe planned summary measures, methods of handling data and methods of combining data from studies, including any planned exploration of consistency (such as I^2^, Kendall’s τ) | Quantitative synthesis not applicable |
|  | 15c | Describe any proposed additional analyses (such as sensitivity or subgroup analyses, meta-regression) | Descriptive comparison by setting, intervention type, and delivery platform |
|  | 15d | If quantitative synthesis is not appropriate, describe the type of summary planned | Narrative synthesis with tables and graphs for summarizing findings |
| Meta-bias(es) | 16 | Specify any planned assessment of meta-bias(es) (such as publication bias across studies, selective reporting within studies) | Not applicable to scoping review |
| Confidence in cumulative evidence | 17 | Describe how the strength of the body of evidence will be assessed (such as GRADE) | Not formally assessed; focus is mapping and identifying gaps |

*** It is strongly recommended that this checklist be read in conjunction with the PRISMA-P Explanation and Elaboration (cite when available) for important clarification on the items. Amendments to a review protocol should be tracked and dated. The copyright for PRISMA-P (including checklist) is held by the PRISMA-P Group and is distributed under a Creative Commons Attribution Licence 4.0.**

*From: Shamseer L, Moher D, Clarke M, Ghersi D, Liberati A, Petticrew M, Shekelle P, Stewart L, PRISMA-P Group. Preferred reporting items for systematic review and meta-analysis protocols (PRISMA-P) 2015: elaboration and explanation. BMJ. 2015 Jan 2;349(jan02 1):g7647.*
