# Supplementary material for: Gender-transformative health promotion interventions for linking and retaining tuberculosis-diagnosed adult men in care in sub-Saharan Africa: A scoping review protocol
Source: PLoS One. 2026 Jan 8;21(1):e0339666. doi: 10.1371/journal.pone.0339666 (PMC12782366; doi:10.1371/journal.pone.0339666)
Supplement: S3 Fig — (DOCX) [file pone.0339666.s003.docx]

# Search Strategy — Scoping Review

| **Database** | **Rationale** | **#** | **Search strategy** |
| --- | --- | --- | --- |
| Pubmed (MEDLINE) | Broad coverage of biomedical literature and comprehensive indexing with MeSH. Essential for TB clinical, epidemiological and intervention studies and for retrieving MeSH tagged literature on Africa (e.g. "Africa South of the Sahara"). | #1 | ("Tuberculosis"[MeSH] OR "Mycobacterium tuberculosis"[MeSH] OR tuberculos*[tiab] OR TB[tiab] OR XMTB[tiab]) |
|  |  | #2 | ("Men's Health"[MeSH] OR "Masculinity"[MeSH] OR men[tiab] OR male[tiab] OR masculinity[tiab] OR "gender norms"[tiab] OR "gender roles"[tiab]) |
|  |  | #3 | ("Health Promotion"[MeSH] OR "Health Behavior"[MeSH] OR "Behavioral Research"[MeSH] OR "Health Education"[MeSH] OR "Gender Equity"[MeSH] OR "health equity"[tiab] OR "gender-transformative"[tiab] OR "gender transformative"[tiab] OR "gender-transforming"[tiab] OR "gender-transformative interventions"[tiab] OR "health promotion interventions"[tiab] OR "behavioral interventions"[tiab] OR "behavioural interventions"[tiab]) |
|  |  | #4 | ("Scoping Review"[tiab] OR "scoping review protocol"[tiab] OR "Stop TB"[tiab] OR "TB elimination"[tiab]) |
|  |  | #5 | ("Africa South of the Sahara"[MeSH] OR "Sub-Saharan Africa"[tiab] OR "Sub Saharan Africa"[tiab] OR SSA[tiab] OR "Angola" OR "Benin" OR "Botswana" OR "Burkina Faso" OR "Burundi" OR "Cabo Verde" OR "Cameroon" OR "Central African Republic" OR "Chad" OR "Comoros" OR "Congo, Democratic Republic" OR "Congo, Republic" OR "Côte d’Ivoire" OR "Equatorial Guinea" OR "Eritrea" OR "Eswatini" OR "Ethiopia" OR "Gabon" OR "Gambia" OR "Ghana" OR "Guinea" OR "Guinea-Bissau" OR "Kenya" OR "Lesotho" OR "Liberia" OR "Madagascar" OR "Malawi" OR "Mali" OR "Mauritania" OR "Mauritius" OR "Mozambique" OR "Namibia" OR "Niger" OR "Nigeria" OR "Rwanda" OR "Sao Tome and Principe" OR "Senegal" OR "Seychelles" OR "Sierra Leone" OR "Somalia" OR "South Africa" OR "South Sudan" OR "Sudan" OR "Tanzania" OR "Togo" OR "Uganda" OR "Zambia" OR "Zimbabwe") |
|  |  | #6 | #1 AND (#2 OR #3 OR #4) AND #5 |
| Cochrane Library Search | High quality evidence synthesis and trials registers. Useful to identify randomized trials, systematic reviews, and protocols relevant to behavioral and health promotion interventions. | #1 | MeSH descriptor: [Tuberculosis] explode all trees |
|  |  | #2 | (tuberculos* OR TB OR XMTB):ti,ab,kw |
|  |  | #3 | #1 OR #2 |
|  |  | #4 | MeSH descriptor: [Men's Health] explode all trees |
|  |  | #5 | MeSH descriptor: [Masculinity] explode all trees |
|  |  | #6 | (men OR male OR masculinity OR "gender norms" OR "gender roles"):ti,ab,kw |
|  |  | #7 | #4 OR #5 OR #6 |
|  |  | #8 | MeSH descriptor: [Health Promotion] explode all trees |
|  |  | #9 | MeSH descriptor: [Health Behavior] explode all trees |
|  |  | #10 | ("gender-transformative" OR "gender-transformative interventions" OR "health promotion interventions" OR "behavioral interventions" OR "health equity"):ti,ab,kw |
|  |  | #11 | #8 OR #9 OR #10 |
|  |  | #12 | ("Scoping review" OR "scoping review protocol" OR "Stop TB" OR "TB elimination"):ti,ab,kw |
|  |  | #13 | ("sub-saharan africa" OR "sub saharan africa" OR SSA):ti,ab,kw OR MeSH descriptor: [Africa South of the Sahara] explode all trees OR "Angola" OR "Benin" OR "Botswana" OR "Burkina Faso" OR "Burundi" OR "Cabo Verde" OR "Cameroon" OR "Central African Republic" OR "Chad" OR "Comoros" OR "Congo, Democratic Republic" OR "Congo, Republic" OR "Côte d’Ivoire" OR "Equatorial Guinea" OR "Eritrea" OR "Eswatini" OR "Ethiopia" OR "Gabon" OR "Gambia" OR "Ghana" OR "Guinea" OR "Guinea-Bissau" OR "Kenya" OR "Lesotho" OR "Liberia" OR "Madagascar" OR "Malawi" OR "Mali" OR "Mauritania" OR "Mauritius" OR "Mozambique" OR "Namibia" OR "Niger" OR "Nigeria" OR "Rwanda" OR "Sao Tome and Principe" OR "Senegal" OR "Seychelles" OR "Sierra Leone" OR "Somalia" OR "South Africa" OR "South Sudan" OR "Sudan" OR "Tanzania" OR "Togo" OR "Uganda" OR "Zambia" OR "Zimbabwe" |
|  |  | #14 | #3 AND (#7 OR #11 OR #12) AND #13 |
| Embase.com | Stronger European and conference-record coverage and additional indexing (Emtree). Captures studies not in MEDLINE and improves retrieval of intervention and public health literature. | #1 | 'tuberculosis'/exp OR (tuberculos* OR TB OR XMTB):ti,ab,kw |
|  |  | #2 | 'men's health'/exp OR 'masculinity'/exp OR (men OR male OR masculinity OR "gender norms" OR "gender roles"):ti,ab,kw |
|  |  | #3 | 'health promotion'/exp OR 'health behavior'/exp OR 'behavioral research'/exp OR 'gender equity'/exp OR 'health equity'/exp OR ("gender-transformative" OR "gender-transformative interventions" OR "health promotion interventions" OR "behavioral interventions" OR "behavioural interventions"):ti,ab,kw |
|  |  | #4 | ("Stop TB" OR "TB elimination"):ti,ab,kw |
|  |  | #5 | ('scoping review'/exp OR 'review'/exp OR "scoping review protocol":ti,ab) |
|  |  | #6 | ( "sub-saharan africa" OR "sub saharan africa" OR SSA OR 'africa south of the sahara'/exp OR "Angola" OR "Benin" OR "Botswana" OR "Burkina Faso" OR "Burundi" OR "Cabo Verde" OR "Cameroon" OR "Central African Republic" OR "Chad" OR "Comoros" OR "Congo, Democratic Republic" OR "Congo, Republic" OR "Côte d’Ivoire" OR "Equatorial Guinea" OR "Eritrea" OR "Eswatini" OR "Ethiopia" OR "Gabon" OR "Gambia" OR "Ghana" OR "Guinea" OR "Guinea-Bissau" OR "Kenya" OR "Lesotho" OR "Liberia" OR "Madagascar" OR "Malawi" OR "Mali" OR "Mauritania" OR "Mauritius" OR "Mozambique" OR "Namibia" OR "Niger" OR "Nigeria" OR "Rwanda" OR "Sao Tome and Principe" OR "Senegal" OR "Seychelles" OR "Sierra Leone" OR "Somalia" OR "South Africa" OR "South Sudan" OR "Sudan" OR "Tanzania" OR "Togo" OR "Uganda" OR "Zambia" OR "Zimbabwe" ):ti,ab,kw |
|  |  | #7 | #1 AND (#2 OR #3 OR #4 OR #5) AND #6 |
| Global Health Search Strategy | Focused on public health literature and international/grey literature that may not be indexed in biomedical databases. Valuable for policy reports and country/regional TB program evaluations in Africa. | #1 | exp tuberculosis/ OR (tuberculos* OR TB OR XMTB).ti,ab |
|  |  | #2 | exp men's health/ OR exp masculinity/ OR (men OR male OR masculinity OR "gender norms" OR "gender roles").ti,ab |
|  |  | #3 | exp health promotion/ OR exp behavioral intervention/ OR ("gender-transformative" OR "gender-transformative interventions" OR "health promotion interventions" OR "behavioral interventions" OR "health equity"):ti,ab |
|  |  | #4 | ("Stop TB" OR "TB elimination").ti,ab |
|  |  | #5 | exp scoping review/ OR ("scoping review protocol").ti,ab |
|  |  | #6 | ("sub-saharan africa" OR "sub saharan africa" OR SSA OR exp africa south of the sahara/ OR "Angola" OR "Benin" OR "Botswana" OR "Burkina Faso" OR "Burundi" OR "Cabo Verde" OR "Cameroon" OR "Central African Republic" OR "Chad" OR "Comoros" OR "Congo, Democratic Republic" OR "Congo, Republic" OR "Côte d’Ivoire" OR "Equatorial Guinea" OR "Eritrea" OR "Eswatini" OR "Ethiopia" OR "Gabon" OR "Gambia" OR "Ghana" OR "Guinea" OR "Guinea-Bissau" OR "Kenya" OR "Lesotho" OR "Liberia" OR "Madagascar" OR "Malawi" OR "Mali" OR "Mauritania" OR "Mauritius" OR "Mozambique" OR "Namibia" OR "Niger" OR "Nigeria" OR "Rwanda" OR "Sao Tome and Principe" OR "Senegal" OR "Seychelles" OR "Sierra Leone" OR "Somalia" OR "South Africa" OR "South Sudan" OR "Sudan" OR "Tanzania" OR "Togo" OR "Uganda" OR "Zambia" OR "Zimbabwe") |
|  |  | #7 | 1 AND (2 OR 3 OR 4 OR 5) AND 6 |
| Web of Science Search Strategy | Multidisciplinary, citation‑linking database that helps identify influential and highly cited studies, track citations, and capture literature from diverse journals not indexed elsewhere. | #1 | TS=(tuberculosis OR TB OR XMTB) AND TS=(men OR male OR masculinity OR "gender norms" OR "gender roles") AND TS=("gender-transformative" OR "gender-transformative interventions" OR "health promotion interventions" OR "behavioral interventions" OR "health equity") AND TS=("End TB Strategy 2035" OR "End TB Strategy" OR "Stop TB" OR "TB elimination" OR "scoping review" OR "scoping review protocol") AND TS=("sub-saharan africa" OR "sub saharan africa" OR SSA OR "Angola" OR "Benin" OR "Botswana" OR "Burkina Faso" OR "Burundi" OR "Cabo Verde" OR "Cameroon" OR "Central African Republic" OR "Chad" OR "Comoros" OR "Congo, Democratic Republic" OR "Congo, Republic" OR "Côte d’Ivoire" OR "Equatorial Guinea" OR "Eritrea" OR "Eswatini" OR "Ethiopia" OR "Gabon" OR "Gambia" OR "Ghana" OR "Guinea" OR "Guinea-Bissau" OR "Kenya" OR "Lesotho" OR "Liberia" OR "Madagascar" OR "Malawi" OR "Mali" OR "Mauritania" OR "Mauritius" OR "Mozambique" OR "Namibia" OR "Niger" OR "Nigeria" OR "Rwanda" OR "Sao Tome and Principe" OR "Senegal" OR "Seychelles" OR "Sierra Leone" OR "Somalia" OR "South Africa" OR "South Sudan" OR "Sudan" OR "Tanzania" OR "Togo" OR "Uganda" OR "Zambia" OR "Zimbabwe") |
| PsycINFO Search Strategy | Source for psychological, behavioral and social science literature. Important for retrieving studies on masculinity, gender norms, behavior change and intervention acceptability. | #1 | DE "Tuberculosis" OR (tuberculos* OR TB OR XMTB):ti,ab |
|  |  | #2 | DE "Men's Health" OR DE "Masculinity" OR (men OR male OR masculinity OR "gender norms" OR "gender roles"):ti,ab |
|  |  | #3 | DE "Health Promotion" OR DE "Behavioral Interventions" OR ("gender-transformative" OR "gender-transformative interventions" OR "health promotion interventions" OR "behavioral interventions" OR "health equity"):ti,ab |
|  |  | #4 | ("Stop TB" OR "TB elimination" OR "scoping review" OR "scoping review protocol"):ti,ab |
|  |  | #5 | ("sub-saharan africa" OR "sub saharan africa" OR SSA OR DE "Africa South of the Sahara" OR "Angola" OR "Benin" OR "Botswana" OR "Burkina Faso" OR "Burundi" OR "Cabo Verde" OR "Cameroon" OR "Central African Republic" OR "Chad" OR "Comoros" OR "Congo, Democratic Republic" OR "Congo, Republic" OR "Côte d’Ivoire" OR "Equatorial Guinea" OR "Eritrea" OR "Eswatini" OR "Ethiopia" OR "Gabon" OR "Gambia" OR "Ghana" OR "Guinea" OR "Guinea-Bissau" OR "Kenya" OR "Lesotho" OR "Liberia" OR "Madagascar" OR "Malawi" OR "Mali" OR "Mauritania" OR "Mauritius" OR "Mozambique" OR "Namibia" OR "Niger" OR "Nigeria" OR "Rwanda" OR "Sao Tome and Principe" OR "Senegal" OR "Seychelles" OR "Sierra Leone" OR "Somalia" OR "South Africa" OR "South Sudan" OR "Sudan" OR "Tanzania" OR "Togo" OR "Uganda" OR "Zambia" OR "Zimbabwe") |
|  |  | #6 | S1 AND (S2 OR S3 OR S4) AND S5 |
| Google Scholar Search Strategy | Broad, inclusive search of peer‑reviewed and grey literature (theses, reports, conference papers). Useful to capture non‑indexed publications and to follow citation chains; however, it requires careful de‑duplication and screening. | #1 | ("tuberculosis" OR "TB") AND (men OR male OR masculinity OR "gender norms" OR "gender roles") AND ("gender-transformative" OR "gender-transformative interventions" OR "health promotion interventions" OR "behavioral interventions" OR "health equity") AND ("End TB Strategy 2035" OR "End TB Strategy" OR "Stop TB" OR "TB elimination" OR "scoping review" OR "scoping review protocol") AND ("sub-saharan africa" OR "sub saharan africa" OR SSA OR "Angola" OR "Benin" OR "Botswana" OR "Burkina Faso" OR "Burundi" OR "Cabo Verde" OR "Cameroon" OR "Central African Republic" OR "Chad" OR "Comoros" OR "Congo, Democratic Republic" OR "Congo, Republic" OR "Côte d’Ivoire" OR "Equatorial Guinea" OR "Eritrea" OR "Eswatini" OR "Ethiopia" OR "Gabon" OR "Gambia" OR "Ghana" OR "Guinea" OR "Guinea-Bissau" OR "Kenya" OR "Lesotho" OR "Liberia" OR "Madagascar" OR "Malawi" OR "Mali" OR "Mauritania" OR "Mauritius" OR "Mozambique" OR "Namibia" OR "Niger" OR "Nigeria" OR "Rwanda" OR "Sao Tome and Principe" OR "Senegal" OR "Seychelles" OR "Sierra Leone" OR "Somalia" OR "South Africa" OR "South Sudan" OR "Sudan" OR "Tanzania" OR "Togo" OR "Uganda" OR "Zambia" OR "Zimbabwe") |
| Africa‑Wide Information Search Strategy | Regional bibliographic database that indexes African journals and grey literature often missed by global databases. Critical for locating locally published studies, program reports, and context‑specific evidence from Sub‑Saharan Africa. | #1 | (tuberculos* OR TB OR XMTB):ti,ab |
|  |  | #2 | (men OR male OR masculinity OR "gender norms" OR "gender roles"):ti,ab |
|  |  | #3 | ("gender-transformative" OR "gender-transformative interventions" OR "health promotion interventions" OR "behavioral interventions" OR "health equity"):ti,ab |
|  |  | #4 | ("Stop TB" OR "TB elimination"):ti,ab |
|  |  | #5 | ("scoping review" OR "scoping review protocol"):ti,ab |
|  |  | #6 | ("sub-saharan africa" OR "sub saharan africa" OR SSA OR "Angola" OR "Benin" OR "Botswana" OR "Burkina Faso" OR "Burundi" OR "Cabo Verde" OR "Cameroon" OR "Central African Republic" OR "Chad" OR "Comoros" OR "Congo, Democratic Republic" OR "Congo, Republic" OR "Côte d’Ivoire" OR "Equatorial Guinea" OR "Eritrea" OR "Eswatini" OR "Ethiopia" OR "Gabon" OR "Gambia" OR "Ghana" OR "Guinea" OR "Guinea-Bissau" OR "Kenya" OR "Lesotho" OR "Liberia" OR "Madagascar" OR "Malawi" OR "Mali" OR "Mauritania" OR "Mauritius" OR "Mozambique" OR "Namibia" OR "Niger" OR "Nigeria" OR "Rwanda" OR "Sao Tome and Principe" OR "Senegal" OR "Seychelles" OR "Sierra Leone" OR "Somalia" OR "South Africa" OR "South Sudan" OR "Sudan" OR "Tanzania" OR "Togo" OR "Uganda" OR "Zambia" OR "Zimbabwe"):ti,ab |
|  |  | #7 | S1 AND (S2 OR S3 OR S4 OR S5) AND S6 |
| WHO Library / Global Index Medicus Search Strategy | Aggregates regional indexes (AFRO, EMRO, WPR, etc.) and includes national reports and WHO documents. Essential for public health policy documents, surveillance reports, and regional TB strategy materials relevant to Sub‑Saharan Africa. | #1 | (tuberculosis OR TB OR XMTB) AND (men OR male OR masculinity OR "gender norms" OR "gender roles") AND ("gender-transformative" OR "gender-transformative interventions" OR "health promotion interventions" OR "behavioral interventions" OR "health equity") AND ("TB elimination" OR "scoping review" OR "scoping review protocol") AND ("sub-saharan africa" OR "sub saharan africa" OR SSA OR "Angola" OR "Benin" OR "Botswana" OR "Burkina Faso" OR "Burundi" OR "Cabo Verde" OR "Cameroon" OR "Central African Republic" OR "Chad" OR "Comoros" OR "Congo, Democratic Republic" OR "Congo, Republic" OR "Côte d’Ivoire" OR "Equatorial Guinea" OR "Eritrea" OR "Eswatini" OR "Ethiopia" OR "Gabon" OR "Gambia" OR "Ghana" OR "Guinea" OR "Guinea-Bissau" OR "Kenya" OR "Lesotho" OR "Liberia" OR "Madagascar" OR "Malawi" OR "Mali" OR "Mauritania" OR "Mauritius" OR "Mozambique" OR "Namibia" OR "Niger" OR "Nigeria" OR "Rwanda" OR "Sao Tome and Principe" OR "Senegal" OR "Seychelles" OR "Sierra Leone" OR "Somalia" OR "South Africa" OR "South Sudan" OR "Sudan" OR "Tanzania" OR "Togo" OR "Uganda" OR "Zambia" OR "Zimbabwe") |
