# Supplementary material for: Gender-transformative health promotion interventions for linking and retaining tuberculosis-diagnosed adult men in care in sub-Saharan Africa: A scoping review protocol
Source: PLoS One. 2026 Jan 8;21(1):e0339666. doi: 10.1371/journal.pone.0339666 (PMC12782366; doi:10.1371/journal.pone.0339666)
Supplement: S4 Fig — (DOCX) [file pone.0339666.s004.docx]

**Data charting tool for scoping review of gender-transformative health promotion TB interventions**

| **Category** | **Data to be Extracted / Charted** | **Example or Guidance** |
| --- | --- | --- |
| **Study Identification** | Author(s); Year; Title; Journal / Source | Moyo et al., 2022, *BMC Public Health* |
| **Study Location & Context** | Country; Region; Setting (urban/rural); Level of care (community clinic, hospital, specialized TB centre, etc.) | South Africa; Urban clinic; Specialized TB centre |
| **Setting Characteristics** | Where TB was diagnosed and treated; Care providers responsible for TB management (specialist vs generalist staff) | Diagnosed at community clinic; Managed by generalist nurses |
| **Policy & Governance Environment** | Presence of anti-discrimination policies; Presence of gender-sensitive TB policies; Evidence of enforcement (inspection reports, audits) | Anti-discrimination policy present but weak enforcement based on facility audit |
| **Study Aims / Objectives** | Main purpose or research questions | To understand how gender norms influence men’s TB care-seeking practices |
| **Study Design / Methodology** | Qualitative, quantitative, mixed; study design type (cross-sectional, RCT, cohort, etc.) | Mixed-methods community study |
| **Study Population** | Gender; Age range; Sample size; Target group | Adult men (18–49), n=132 |
| **Type of TB Assessed** | Pulmonary/Extra-pulmonary; DR-TB vs drug-sensitive TB | Pulmonary drug-sensitive TB |
| **TB Treatment Scheme & Duration** | Treatment regimen; Length of treatment; Use of Directly Observed Therapy (DOTS) or alternatives | 6-month standard regimen; clinic-based DOTS |
| **Complementary / Alternative Medicines** | Whether CAMs were included in treatment; Type and rationale | Herbal remedies encouraged by community leaders |
| **Direct & Indirect Costs of TB Care** | Transport, food, income loss, user-fees, etc. | Out-of-pocket travel and lost wages during clinic visits |
| **Funder of TB Treatment** | Government, donor funding, insurance, out-of-pocket | Government subsidized medications; patients pay for transport |
| **Intervention Type / Approach** | Description of intervention; Preventive or curative; Gender-specific or not; Cultural tailoring; Community engagement | Peer-led men’s support circles with community health workers |
| **Gender-Transformative Elements** | How norms, roles, power relations were questioned or reshaped | Challenged harmful masculinity norms that delayed health seeking |
| **Health Promotion Strategies Used** | Education, peer advocacy, media, workplace outreach, community theatre, etc. | Theatre-based TB awareness shows |
| **Theoretical / Conceptual Framework** | Model/framework guiding intervention | Gender-Transformative Health Promotion Framework |
| **Duration of Participant Follow-Up** | Timeframe; Frequency of follow-up contact | 6-month follow-up during treatment |
| **Reasons for End of Follow-Up** | Completion, loss to follow-up, transfer, withdrawal, death | Follow-up ended due to treatment completion |
| **Level of Adherence to TB Treatment** | Measures of adherence (qualitative or quantitative) | 82% completion rate |
| **Reasons for Stopping TB Treatment** | E.g. stigma, death, side effects, work conflicts, migration | Defaulted due to employment demands |
| **Key Findings / Outcomes** | Qualitative themes or quantitative outcomes (clinical, psychosocial, economic) | Higher treatment adherence and improved confidence in seeking care |
| **Participant Outcomes Beyond Clinical** | Social role changes, livelihoods, wellbeing, quality of life | Improved family communication and social reintegration |
| **Barriers & Facilitators** | To TB treatment engagement and/or the health promotion intervention | Barriers: stigma, clinic wait times. Facilitators: male peer mentors |
| **Authors’ Conclusions** | Summary of research team’s interpretation | Gender-responsive approaches supported better engagement in care |
| **Quality Appraisal (if applicable)** | MMAT or other tool summary | 4/5 “Yes” ratings |
| **Reviewer Notes / Relevance** | Notes on fit with review question; contextual observations | Strong example of community-grounded, masculinity-aware intervention |

**Instructions for Use**

- Chart data consistently and precisely across all included studies.
- Capture both numerical outcomes (e.g., % adherence) and qualitative insights (e.g., norms, stigma).
- Pilot the chart on 2-3 studies to confirm usability.
