# Supplementary material for: Gender-transformative health promotion interventions for linking and retaining tuberculosis-diagnosed adult men in care in sub-Saharan Africa: A scoping review protocol
Source: PLoS One. 2026 Jan 8;21(1):e0339666. doi: 10.1371/journal.pone.0339666 (PMC12782366; doi:10.1371/journal.pone.0339666)
Supplement: S6 Table — (DOCX) [file pone.0339666.s006.docx]

| **Country** | **World Bank 2025 Income Classification** |
| --- | --- |
| Angola | Lower middle income |
| Benin | Lower middle income |
| Botswana | Upper middle income |
| Burkina Faso | Low income |
| Burundi | Low income |
| Cameroon | Lower middle income |
| Cape Verde | Lower middle income |
| Central African Republic | Low income |
| Chad | Low income |
| Comoros | Lower middle income |
| Congo, Rep. | Lower middle income |
| Congo, Dem. Rep. | Low income |
| Côte d’Ivoire | Lower middle income |
| Djibouti | Lower middle income |
| Equatorial Guinea | Upper middle income |
| Eritrea | Low income |
| Eswatini | Lower middle income |
| Ethiopia | Low income |
| Gabon | Upper middle income |
| Gambia, The | Low income |
| Ghana | Lower middle income |
| Guinea | Lower middle income |
| Guinea-Bissau | Low income |
| Kenya | Lower middle income |
| Lesotho | Lower middle income |
| Liberia | Low income |
| Madagascar | Low income |
| Malawi | Low income |
| Mali | Low income |
| Mauritania | Lower middle income |
| Mauritius | Upper middle income |
| Mozambique | Low income |
| Namibia | Upper middle income |
| Niger | Low income |
| Nigeria | Lower middle income |
| Rwanda | Low income |
| Sao Tome & Príncipe | Lower middle income |
| Senegal | Lower middle income |
| Sierra Leone | Low income |
| Somalia | Low income |
| South Sudan | Low income |
| Sudan | Low income |
| Tanzania | Lower middle income |
| Togo | Low income |
| Uganda | Low income |
| Zambia | Lower middle income |
| Zimbabwe | Lower middle income |
