# Supplementary material for: Gender-transformative health promotion interventions for linking and retaining tuberculosis-diagnosed adult men in care in sub-Saharan Africa: A scoping review protocol
Source: PLoS One. 2026 Jan 8;21(1):e0339666. doi: 10.1371/journal.pone.0339666 (PMC12782366; doi:10.1371/journal.pone.0339666)
Supplement: Attachment — Submitted filename: PONE-D-25-36889_reviewed by Mickael Essouma.pdf [file pone.0339666.s007.pdf]

PLOS ONE

Gender-transformative and health promotion interventions for linking and retaining tuberculosis diagnosed adult men (18 years and above) in care in sub Saharan Africa: A scoping review protocol

--Manuscript Draft--

**Manuscript Number:**	PONE-D-25-36889
**Article Type:**	Study Protocol
**Full Title:**	Gender-transformative and health promotion interventions for linking and retaining tuberculosis diagnosed adult men (18 years and above) in care in sub Saharan Africa: A scoping review protocol
**Short Title:**	A Scoping review protocol for gender-transformative tuberculosis interventions for men in sub-Saharan Africa
**Corresponding Author:**	Siyabonga Kave, PhD<br>University of Kwazulu-Natal<br>Durban, KwaZulu-Natal SOUTH AFRICA
**Keywords:**	Tuberculosis, gender-transformative interventions, men's health, linkage to care, retention in care, health promotion, sub-Saharan Africa, scoping review.
**Abstract:**	Background<br>Tuberculosis (TB) remains one of the leading causes of morbidity and mortality globally, with men disproportionately affected. In sub-Saharan Africa (SSA), gender disparities in TB care persist, as adult men are less likely to link to care, initiate treatment, or remain engaged in long-term care. Social and structural determinants—particularly gender norms and masculinities—critically shape men's health-seeking behaviours. Despite global commitments to gender equity in health, many TB programs remain gender-blind or neutral, with limited application of gender-transformative and health promotion interventions tailored to men's specific needs.<br>Purpose of the Scoping Review<br>This scoping review aims to map existing evidence on gender-transformative and health promotion interventions designed to strengthen linkage to and retention in care for adult men (≥18 years) diagnosed with TB in SSA. The central research question is: What evidence exists on gender-transformative and health promotion interventions aimed at improving linkage to and retention in care among adult men diagnosed with TB in sub-Saharan Africa?<br>Methods<br>The review will follow Arksey and O'Malley's five-stage scoping review framework, with guidance from the PRISMA-ScR checklist. A comprehensive literature search will be conducted across PubMed, Web of Science, MEDLINE, PsychINFO, and grey literature sources. Eligible studies will include those published between 2015 and 2024 in English, focused on adult men diagnosed with TB in SSA. Data will be extracted and analyzed through narrative synthesis. The quality of included studies will be assessed using the Mixed Methods Appraisal Tool (MMAT) 2018.<br>As of July 2025, the protocol has been finalized and piloting of database search strategies is underway. Title and abstract screening will begin in August 2025, with full completion expected by September 2025. Full-text screening and data extraction will take place between October and November 2025. Analysis and synthesis of findings will be conducted in December 2025, and we anticipate submitting the final manuscript for publication by January 2026.<br>Discussion<br>This review will provide a comprehensive overview of gender-transformative and health promotion interventions aimed at improving TB care outcomes for men in SSA. It will identify core intervention components, delivery platforms, and implementation contexts, as well as critical gaps in current approaches. Findings will inform the co-development of culturally responsive, gender-sensitive strategies that enhance men's engagement in TB care. Ultimately, the review contributes to strengthening TB programming aligned with the WHO End TB Strategy's 2035 goals.<br>Registration: Open Science Framework.<br>https://doi.org/10.17605/OSF.IO/RNF2TLicense: CC0 1.0 Universal

Powered by Editorial Manager® and ProduXion Manager® from Aries Systems Corporation

Order of Authors:	Siyabonga Kave, PhD
	Yandisa Sikweyiya, PhD in Public Health
	Nelisiwe Khuzwayo, PhD Public Health
Opposed Reviewers:	
Additional Information:	
**Question**	**Response**
**Financial Disclosure**<br><br>Enter a financial disclosure statement that describes the sources of funding for the work included in this submission. Review the submission guidelines for detailed requirements. View published research articles from *PLOS ONE* for specific examples.<br><br>This statement is required for submission and **will appear in the published article** if the submission is accepted. Please make sure it is accurate.<br><br>**Funded studies**<br>Enter a statement with the following details:<br>• Initials of the authors who received each award<br>• Grant numbers awarded to each author<br>• The full name of each funder<br>• URL of each funder website<br>• Did the sponsors or funders play any role in the study design, data collection and analysis, decision to publish, or preparation of the manuscript?<br><br>Did you receive funding for this work?	The author(s) received no specific funding for this work.
**Competing Interests**<br><br>Use the instructions below to enter a competing interest statement for this submission. On behalf of all authors, disclose any competing interests that could be perceived to bias this work—acknowledging all financial support and any other relevant financial or non-financial competing interests.	I have read the journal's policy and the authors of this manuscript have no competing interest

This statement is required for submission and **will appear in the published article** if the submission is accepted. Please make sure it is accurate and that any funding sources listed in your Funding Information later in the submission form are also declared in your Financial Disclosure statement.<br><br>View published research articles from *PLOS ONE* for specific examples.<br><br>**NO authors have competing interests**<br><br>Enter: *The authors have declared that no competing interests exist.*<br><br>**Authors with competing interests**<br><br>Enter competing interest details beginning with this statement:<br><br>*I have read the journal's policy and the authors of this manuscript have the following competing interests: [insert competing interests here]*<br><br>* typeset	
**Ethics Statement**<br><br>Enter an ethics statement for this submission. This statement is required if the study involved:<br><br>• Human participants<br>• Human specimens or tissue<br>• Vertebrate animals or cephalopods<br>• Vertebrate embryos or tissues<br>• Field research<br><br>Write "N/A" if the submission does not require an ethics statement.<br><br>General guidance is provided below. Consult the submission guidelines for detailed instructions. **Make sure that all information entered here is included in the Methods section of the manuscript.**	This scoping review does not involve the collection of primary data from human participants. All data sources to be included are publicly available and published in peer-reviewed journals or grey literature. Therefore, ethical approval is not required for this study.

Powered by Editorial Manager® and ProduXion Manager® from Aries Systems Corporation

**Format for specific study types**<br><br>**Human Subject Research (involving human participants and/or tissue)**<br>• Give the name of the institutional review board or ethics committee that approved the study<br>• Include the approval number and/or a statement indicating approval of this research<br>• Indicate the form of consent obtained (written/oral) or the reason that consent was not obtained (e.g. the data were analyzed anonymously)<br><br>**Animal Research (involving vertebrate animals, embryos or tissues)**<br>• Provide the name of the Institutional Animal Care and Use Committee (IACUC) or other relevant ethics board that reviewed the study protocol, and indicate whether they approved this research or granted a formal waiver of ethical approval<br>• Include an approval number if one was obtained<br>• If the study involved *non-human primates*, add *additional details* about animal welfare and steps taken to ameliorate suffering<br>• If anesthesia, euthanasia, or any kind of animal sacrifice is part of the study, include briefly which substances and/or methods were applied<br><br>**Field Research**<br><br>Include the following details if this study involves the collection of plant, animal, or other materials from a natural setting:<br>• Field permit number<br>• Name of the institution or relevant body that granted permission	
**Data Availability**<br><br>Authors are required to make the data underlying their research findings fully available, without restriction.<br><br>If the manuscript reports pilot or preliminary results, the data underlying those results must be made available at	N/A - No results are reported

Powered by Editorial Manager® and ProduXion Manager® from Aries Systems Corporation

the time of publication. A Data Availability Statement describing where the data can be found is required at submission. Your answers to this question constitute the Data Availability Statement and will be published in the article, if accepted. When legal or ethical requirements prohibit public data sharing, authors must indicate how researchers may obtain access to the data. See the PLOS Data Policy and FAQ for detailed information.<br><br>**Important:** Stating 'data available on request from the author' is not sufficient. If your data are only available upon request, select 'No' for the first question and explain your situation in the text box.<br><br>For protocols without pilot or preliminary data, authors are strongly encouraged to state how they plan to share research data from their study when it is completed or published.<br><br>If your study includes pilot data, will they be made fully available without restriction?	
**Describe where the data may be found in full sentences. If you are copying our sample text, replace any instances of XXX with the appropriate details.**<br><br>• If **no pilot data are reported** in the manuscript, please enter the following: *No datasets were generated or analysed during the current study. All relevant data from this study will be made available upon study completion.*<br>• If the manuscript reports pilot data **held in a public repository**, include URLs, accession numbers or DOIs. If this information will only be available after acceptance, indicate this by ticking the box below. For example: *All XXX files are available from the XXX database (accession number(s) XXX, XXX.).*<br>• If the manuscript reports pilot data	Our study will use secondary data which will be made available when the study is completed and published.

Powered by Editorial Manager® and ProduXion Manager® from Aries Systems Corporation

contained **within the manuscript and/or Supporting Information files**, enter the following:<br>*All relevant data are within the manuscript and its Supporting Information files.*<br>• If neither of these applies but you are able to provide **details of access elsewhere**, with or without limitations, please do so. For example:<br><br>*Data cannot be shared publicly because of [XXX]. Data are available from the XXX Institutional Data Access / Ethics Committee (contact via XXX) for researchers who meet the criteria for access to confidential data.*<br><br>*The data underlying the results presented in the study are available from (include the name of the third party and contact information or URL).*<br>• This text is appropriate if the data are owned by a third party and authors do not have permission to share the data.<br><br>For study protocols with data management or sharing plans, authors are encouraged to briefly describe in their Data Availability Statement how data they generate will be made accessible when the study is completed. For example:<br>• Deidentified research data will be made publicly available when the study is completed and published.<br><br>* typeset	
Additional data availability information:	Tick here if the URLs/accession numbers/DOIs will be available only after acceptance of the manuscript for publication so that we can ensure their inclusion before publication.

Powered by Editorial Manager® and ProduXion Manager® from Aries Systems Corporation

Siyabonga Kave
University of KwaZulu-Natal
School of Nursing and Public Health
Durban, South Africa
siyakave@gmail.com
June 16, 2025

Editor-in-Chief
PLOSONE
Public Library of Science

Re: Submission of Manuscript – Gender-transformative and health promotion interventions for linking and retaining tuberculosis diagnosed adult men (18 years and above) in care in sub-Saharan Africa: A scoping review protocol.

Dear Editor,

I am pleased to submit our manuscript titled **"Gender-transformative and health promotion interventions for linking and retaining tuberculosis diagnosed adult men (18 years and above) in care in sub-Saharan Africa: A scoping review protocol"** for consideration for publication in *PLOS ONE*.

Tuberculosis (TB) continues to be a significant public health issue, especially in sub-Saharan Africa, where adult men face greater obstacles in accessing and staying in TB care. This scoping review protocol describes a systematic and transparent method to map existing evidence on gender-sensitive and health promotion interventions aimed at enhancing linkage to and retention in TB care for adult men in this region.

The review will adhere to the Arksey and O'Malley framework, enhanced by updates from the Joanna Briggs Institute. It features a comprehensive search strategy, well-defined inclusion criteria, and a clear data analysis plan. As of submission, data extraction has not yet been completed, in accordance with PLOS ONE's guidelines for research protocol submissions. We respectfully request that this manuscript be considered as a research protocol in line with PLOS ONE's submission requirements and scope.

This protocol has been registered with the Open Science Framework (DOI: https://doi.org/10.17605/OSF.IO/RNF2T) and is shared under the CC0 1.0 Universal license.

We believe this work is well-aligned with PLOS ONE's mission to disseminate rigorous and inclusive research and will be of interest to readers engaged in global health, TB care, gender equity, and health promotion.

Thank you for considering our manuscript. We look forward to your feedback and hope for a favourable review.

Sincerely,
S.Kave
On behalf of all co-authors

Manuscript

Click here to access/download;Manuscript;Gender-transformative TB care for men in sub-Saharan Africa.docx

Gender-transformative and health promotion interventions for linking and retaining tuberculosis diagnosed adult men (18 years and above) in care in sub-Saharan Africa: A scoping review protocol.

Authors: Siyabonga Kave[1], Yandisa Sikweyiya[1,2,3], Nelisiwe Khuzwayo[1].

1. School of Nursing and Public Health, College of Health Sciences, Howard College Campus, University of KwaZulu-Natal, Durban, South Africa

2. Gender and Health Research Unit, South African Medical Research Council, Pretoria, South Africa

3. School of Public Health, University of the Witwatersrand, Johannesburg, South Africa

Corresponding author:

Email address:

School of Nursing and Public Health, University of KwaZulu-Natal

Durban, South Africa

The discipline of Public Health Medicine, School of Nursing and Public Health, University of KwaZulu-Natal, Durban 4041, South Africa

Abstract:

Background: Tuberculosis (TB) remains a major cause of morbidity and mortality globally, with sub-Saharan Africa (SSA) carrying a significant burden. In 2021, the African Region reported 365,000 TB deaths among HIV-negative people, with men accounting for 54% of these fatalities. Despite expanded access to TB services, men are less likely to engage in care due to structural, socio-cultural, and health system-related barriers. Gender norms associated with masculinity, such as stoicism, self-reliance, and reluctance to seek help, negatively affect men's health-seeking behaviour and retention in care.

Objective: This scoping review aims to map and synthesize evidence on gender-transformative and health promotion interventions that support linkage to and retention in TB care among adult men (aged 18 and above) in SSA.

Methods: Following Arksey and O'Malley's five-stage framework and the Population, Concept, and Context (PCC) model, the review will include peer-reviewed articles, grey literature, and policy documents published between 2015 and 2024. Sources will be identified through searches in PubMed, MEDLINE, Web of Science, PsychInfo, and Google Scholar using relevant keywords. Three independent reviewers will screen studies and extract data using a standardized charting tool. Methodological quality will be assessed using the Mixed Methods Appraisal Tool (MMAT), and findings will be synthesized narratively.

Expected Outcomes: The review will describe the types, delivery platforms, and effectiveness of gender-transformative and health promotion interventions targeting men with TB in SSA. It will also identify implementation challenges and research gaps.

Significance: Findings will inform the design of context-specific, gender-responsive TB interventions and guide future research and policy. By addressing gender disparities in TB care access and outcomes, this review aims to contribute to the WHO End TB Strategy goals of reducing TB incidence and mortality by 90% by 2035.

Registration: *Open Science Framework. https://doi.org/10.17605/OSF.IO/RNF2T*

License*: CC0 1.0 Universal*

Keywords: Tuberculosis, gender-transformative interventions, men's health, linkage to care, retention in care, health promotion, sub-Saharan Africa, scoping review.

Strengths and limitations of this study

Strengthens	Weaknesses
The study focuses on gender transformation, which is a critical gap in TB programming.	Potential for limited evidence as there may be a scarcity of published literature specifically addressing gender-transformative interventions for men with TB.
The attention of the study is directed at exploring interventions tailored for a high risk group's (men) linkages to and retention in TB care.	There may be no clear definition of the term gender-transformation, leading to its inconsistent application across studies, which can complicate synthesis and analysis.
The findings of the study will provide practical insights for national TB programmes and international stakeholders for the enhancement of gender-transformative strategies into TB control efforts.	The review is limited to English-language publications; relevant studies published in French, Portuguese, or other regional languages may be excluded, particularly in multilingual sub-Saharan African countries.

Background

Tuberculosis (TB) remains one of the world's deadliest infectious diseases, causing an estimated 1.25 million deaths annually (CDC, 2025) and ranking just below HIV/AIDS in global mortality burden (WHO, 2021). Despite advances in diagnosis and treatment, TB continues to disproportionately affect low-and-middle-income countries (LMICs), particularly those in sub-Saharan Africa (SSA) (Rine et al., 2023; Montgomery et al., 2016).

In 2021, the African Region recorded 365,000 TB deaths among HIV-negative individuals, with men accounting for 54% and women 32% (WHO, 2023). These figures reflect a stark gender disparity in TB outcomes, driven in part by gender and social norms, and construction of masculinities that encourage men's health-seeking avoidance behaviours, reduce diagnosis rates, and undermine treatment retention among men (Hay et al., 2019).

Globally, it has been shown that TB affects more men than women. In 2021, six million men and 3.4 million women developed TB (WHO, 2022). Like other LMICs, South Africa faces a quadruple burden of diseases, which include non-communicable diseases, maternal and child health, violence and injury and HIV and TB (Goswami,2024). In South Africa, the 2018 National TB Prevalence Survey found that pulmonary TB rates among men aged 15 and older were 1.6 times higher than among women (NDoH, 2021; Dhlakama et al., 2022). A multi-country study across 56 nations reported a male-to-female TB incidence ratio of 1.8 (Chikovore et al., 2020).

Scholars have argued that these disparities are shaped not only by biological differences but also by structural and social determinants, including harmful gender norms and masculine identities that discourage men from engaging with health services (Cornell et al., 2015; Colvin, 2019). Moreover, studies have shown that men are less likely to seek TB care promptly, initiate treatment early, or complete it, leading to high rates of loss to follow-up and elevated mortality (MacPherson et al., 2015; Naidoo et al., 2017).

Although TB treatment is highly effective when accessed early and completed (WHO, 2024), men continue to face greater risks of complications and TB-related death (Likoti

et al., 2023; UNAIDS, 2018). In KwaZulu-Natal, South Africa, men experienced public healthcare facilities as feminized spaces, staff as unwelcoming, and that service hours were incompatible with their work and job-seeking schedules—barriers that discouraged their attendance at healthcare facilities and TB treatment adherence (Fleming et al., 2016; Daniels et al., 2021; Chikovore et al., 2024).

These findings highlight the importance of addressing gendered health system barriers in TB control strategies. Although the WHO's End TB Strategy and other global frameworks recognize the need for gender-responsive TB programming, many TB interventions in SSA remain gender-blind or gender-neutral, failing to address the influence of masculinity on care engagement (Medina-Marino, 2025; Sikweyiya et al., 2025).

While most African countries met TB-related Millennium Development Goals (WHO, 2021; WHO, 2023), persistent gaps in the care cascade remain, particularly among men. Socially entrenched ideals that promote stoicism, discourage vulnerability, and limit help-seeking continue to impede timely diagnosis and treatment (Daniels et al., 2021; Mosiwa, 2025).

Gender-transformative interventions defined as strategies that seek to challenge harmful masculine norms, promote positive health behaviours, and foster male-friendly health systems offer a promising avenue for improving TB outcomes among men (Majid et al., 2018; Colvin, 2019). When integrated with health promotion interventions, such approaches can support men to construct health-promoting masculinities, while also empowering men to take control of their health (WHO,2019).

The WHO (1986) defines health promotion as "the process of enabling people to increase control over, and to improve, their health." Health promotion efforts extend beyond individual behaviour change to include the creation of supportive environments that reduce stigma, facilitate access, and promote sustained engagement in care (WHO, 2024).

Emerging evidence shows that co-developing interventions with men enhances their cultural relevance, acceptability, and long-term sustainability (Gibbs et al., 2019; WHO, 2024). Despite this growing evidence largely from the Global North, there

remains limited synthesized evidence on the scope, design, and impact of gender-transformative and health promotion interventions targeting men with TB in SSA. Furthermore, while progress has been made in TB diagnostics and surveillance in high-burden countries such as South Africa, Nigeria, and Zambia (WHO,2023; CDC, 2025), the implementation of gender-sensitive approaches remains weak, particularly in settings where masculinity undermines care-seeking (Sharp et al., 2025).

Emerging evidence indicates that co-developing interventions with men enhances their cultural relevance, acceptability, and long-term sustainability (Gibbs et al., 2019; WHO, 2024). However, much of this evidence originates from the Global North, with limited synthesized evidence available on the scope, design, and impact of gender-transformative and health promotion interventions targeting men diagnosed with TB in sub-Saharan Africa (SSA).

While notable progress has been made in TB diagnostics and surveillance (CDC, 2025), the implementation of gender-sensitive and gender-transformative approaches remains weak, especially in settings where hegemonic masculinity norms undermine men's engagement in care (Sharp et al., 2025). This gap is particularly concerning in SSA, where TB incidence and mortality remain high among men.

Ending the global TB epidemic is a core component of Sustainable Development Goal 3 (SDG 3), which seeks to "ensure healthy lives and promote well-being for all at all ages." Specifically, Target 3.3 aims to end the epidemics of AIDS, TB, malaria, and neglected tropical diseases by 2030 (United Nations, 2015). Achieving this target requires not only biomedical advancements but also inclusive, context-specific strategies that address the social and structural determinants of TB, including gender-based disparities in access, treatment, and outcomes (Banda, 2024; Haji, 2024).

Given the urgency of ending TB by 2035 in line with the WHO's End TB Strategy (WHO, 2024), there is a critical need to examine the landscape of gender-transformative and health promotion strategies tailored specifically for men in SSA. A scoping review is therefore warranted to map existing interventions, explore how they are designed and delivered, evaluate their effectiveness, and identify implementation gaps.

The findings from this review will inform the development of culturally and contextually relevant gender-transformative and health promotion interventions aimed at improving men's engagement in the TB care cascade from diagnosis through to treatment completion. Ultimately, this evidence will support the design of gender-equitable TB responses and contribute meaningfully to national, regional, and global efforts to eliminate TB in alignment with the SDGs.

METHODS

The scoping review aims to synthesize literature on the evidence of gender-transformative and health promotion interventions for linking and retaining adult men in TB care in SSA. This scoping review forms part of a PhD study that has been approved by the Biomedical Research Ethics Committee (BREC) of the University of KwaZulu-Natal (BREC Ref No:00008572/2025).

The scoping review will adopt a five-stage methodological framework as outlined by Arksey and O'Malley (2003). The process includes the following: 1) Identification of the research questions; 2) Identification of relevant studies; 3) Study selection; 4) Charting data; and 4) Collating, summarizing, and reporting findings.

Step1. Identifying the research question

A preliminary literature review was conducted to inform the development of a focused research question for the proposed scoping review. This review aims to systematically map existing gender-transformative health promotion interventions designed to improve linkage to and retention in tuberculosis (TB) care among adult men in sub-Saharan Africa (SSA).

Specifically, the review seeks to examine the nature, design, and delivery of these interventions, as well as to identify gaps in the current evidence base and practice.

The central research question guiding this review is*:* What evidence exists on gender-

transformative and health promotion interventions aimed at strengthening the linkage to and retention in care of adult men diagnosed with TB in sub-Saharan Africa?

After the central research question, there are several sub-questions that the review seeks to explore:

1. Are there any existing gender-transformative health promotion interventions for linking and retaining men diagnosed with TB in care?
2. What are the key design features of gender-transformative interventions that support linkage to and retention in TB care for adult men?
3. Through which platforms are these interventions delivered (e.g., community-based, facility-based, digital, or peer-led approaches)?

Eligibility of the research question

The proposed scoping review seeks to explore the following main research question: What kind of evidence on gender-transformative and health promotion interventions exist to strengthen linkages and retention of men diagnosed with TB in care in SSA? Pollock and colleagues (2023) recommend the use of a Population, Content and Context (PCC) research framework to focus studies to the intended population. Therefore, our eligibility criteria will be guided by the PCC framework to address the research question as follows.

Table 1: Framework for defining the eligibility of the research question.

**Item**	**Description**
Population	Adult men 18 years and above diagnosed with TB, and this will include men who were primary, secondary, lost to follow-up, as well as those who returned to care after having been declared lost to follow-up during treatment.

Concept	Gender-transformative and health promotion interventions to strengthen linkage and retention of men diagnosed with TB. Interventions include Health Education, TB Directly Observed Therapy (DOT), patient or client navigation, and electronic patient records.
Context	Sub-Saharan Africa, Research publications within the last 10 years (2015-2024) and published in English.

Stage 2: Identification of the relevant studies

Relevant literature on men diagnosed with TB will be searched through the review of different sources of information, such as peer-reviewed journal articles. Electronic databases such as PubMed, MEDLINE, Web of Science, PsychInfo, Google Scholar World Health Organization library database, and other websites relevant to the study. In addition, the reference lists of the eligible articles will be analysed for related information.

The principal investigator, together with the primary supervisor and the co-supervisor, will ensure the validity of the search strategy to be used. The search will be confined on articles written in English from 2015 to 2024. In addition, the results of the study will be exported to EndNote library for deduplication and organizing. The study keywords for our research (TB, sub-Saharan Africa, adult men, linkage to care, retention in care, TB diagnosis, health seeking, gender transformative and health promotion intervention) will be used to determine eligibility for inclusion.

Eligibility criteria

The eligibility criteria will feature all the peer-reviewed, published studies that outline the gender-transformative and health promotion interventions that have been used to strengthen linkages and retention of men diagnosed with TB in care in SSA.

The inclusion criteria will be focused on literature as follows: (i) published in English; (ii) within the timeframe of 2015–2024; (iii) conducted in SSA; and (iv) consisting of men diagnosed with TB as a study population. Studies that fail to meet the criteria will be excluded.

The eligibility for the study will be limited to the following criteria:

- Studies on men 18 years and above diagnosed with TB.
- Studies on men diagnosed with TB not engaged in care.
- Studies on men retained in TB care.
- Studies on newly or not yet enrolled on TB treatment.
- Studies on Health Care Worker responsible for managing TB.
- Studies on TB stakeholders responsible for managing the TB programme.
- Studies on TB policy maker
- Studies published from Jan 01, 2015, to Dec 31, 2024.
- Peer-reviewed literature, grey literature, government documents, policy briefs, systematic reviews, and meta-analyses.
- Studies conducted in sub-Saharan African.

Exclusion criteria

Studies that do not meet the eligibility criteria will be excluded:

- Studies published before 2015
- Studies with men diagnosed with TB as a study population but are less than 18 years.

- Studies not conducted in SSA

3. **Selection of eligible studies**

Eligibility criteria will be conducted by three reviewers who will examine the articles and abstracts of studies that have been conducted in SSA. The screening and review of the eligible articles will be done through a two-stage process.

Firstly, the three reviewers will assess eligible articles and abstracts independently from one another using an agreed-upon inclusion criterion as a guide. The second stage of the screening process will be the de-duplication of different databases and the exclusion of ineligible articles and abstracts.

In the event of divergent views and opinions between the reviewers, a mutual agreement will be found through discussions. To ensure that the process is uniformly applied, the following PRISMA flow chart (figure1) will be utilized as a guide:

Figure 1: PRISMA Chart diagram

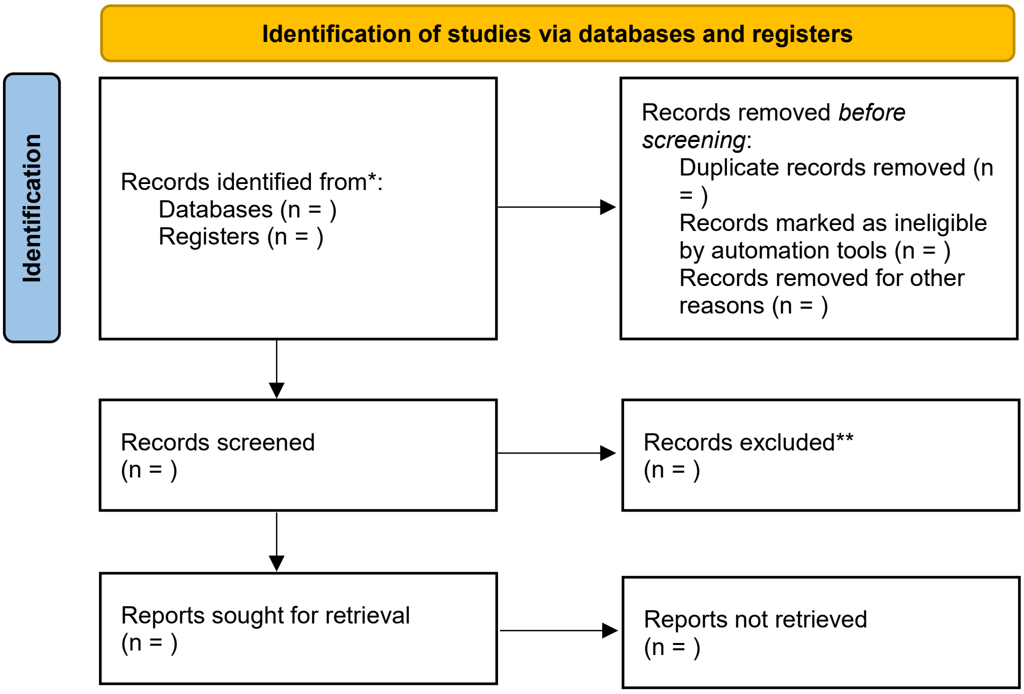

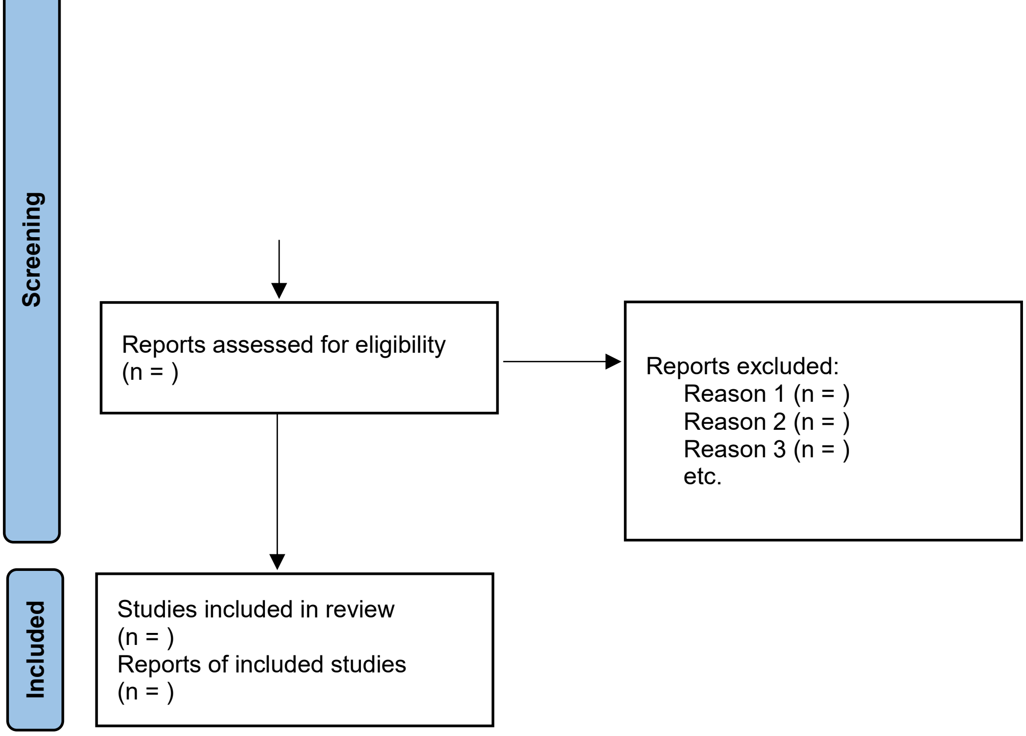

Source: Page MJ, et al. BMJ 2021;372: n71. doi: 10.1136/bmj. n7

Quality assurance of the study

Quality assurance (QA) methods are implemented to ensure that data integrity, quality, and reliability are maintained at every stage of the study (Prasanna et al., 2024; Morrison et al., 2011). The proposed study will use a Mixed Method Appraisal Tool (MMAT) version 2018 (Annexure: S2) (Gavallo et al., 2023). The aim is to provide guidance and adherence to established research protocols (Waghule et al, 2021).

Koneswarekantha and colleagues (2020) argue that there is a need for critical measures to guarantee the accuracy and completeness of the data used to evaluate research outcomes. Therefore, the MMAT tool will be used to assess the methodological quality of the study by examining the meanings of the findings

and their relation to the aim of the study and the implications for future studies.

Stage 4: Charting the data

Data charting is the next stage in the review process (Levac et al, 2010; Peters et al, 2020; Monteiro et al, 2024). Data from all selected articles and abstracts will be extracted in a manner that ensures consistency and reliability. A standardized agreed upon tool (Annexure S4) has been developed by the reviewers to guide the data extraction process. The plan is to collect data from abstracts and selected articles on the type of study and the countries where the study was done.

Relevant information such as the demographic details of human participants (age, gender, health status, socio- economic status), purpose of the study, research design and setting, focal health issue being studied, type of intervention (for interventional studies) and nature of the data gathered from participants will be collected from each article.

To ensure quality, a data extraction framework will be developed to guide the retrieval of the literature consistency with the eligibility criteria of the scoping review. In addition, the three reviewers will conduct a pilot test in a sample of included studies and modify categories if new ones emerge. Any disagreements will be discussed and further specify the inclusion criteria.

5: Collating, summarising, and reporting the result

A narrative data synthesis approach to summarize descriptive data on the gender-transformative and health promotion interventions that have been used to strengthen linkages and retention of men diagnosed with TB in care in SSA will be used. Data will be summarized descriptively with study characteristics presented in tabular and graphical forms will be summarized, interpreted and reported using a narrative approach.

Research gaps will be identified through comparative analysis of the studies and gender transformative health promotion interventions literature and suggest future research topics. In reporting our results, we will highlight similarities and differences in how the gender transformative health promotion interventions have been operationalized in SSA.

Furthermore, the report will provide an overview of the available evidence, aggregate results, and present a summary outline rather than presenting each intervention individually. The proposed scoping review seeks to describe gender-transformative and health promotion interventions that exist to strengthen linkages and retention of men diagnosed with TB in care in SSA?

Therefore, the findings of the scoping review will contribute to the body of literature on interventions that have been studied in SSA aimed at retaining men diagnosed with TB in care. Moreover, our findings will be used towards the development of a gender transformative intervention framework for linkages and retention of men diagnosed with TB. The findings of the study will be disseminated through professional networks, conference presentations and publication in a scientific journal.

Study Status and Timeline

As of July 2025, the protocol has been finalized and piloting of database search strategies is underway. Title and abstract screening will begin in August 2025, with full completion expected by September 2025. Full-text screening and data extraction will take place between October and November 2025. Analysis and synthesis of findings will be conducted in December 2025, and we anticipate submitting the final manuscript for publication by January 2026.

DISCUSSION

The proposed scoping review will map and synthesise existing literature on gender-transformative and health promotion interventions implemented in sub-Saharan Africa (SSA) that aim to strengthen linkage to care and retention among men (18 years and above) diagnosed with TB. This effort aligns directly with the WHO's post-2015 End TB Strategy, which was adopted by the World Health Assembly in 2014 and is embedded within the Sustainable Development Goals (WHO, 2015; Kuupiel et al., 2019).

A core pillar of the End TB Strategy is integrated, patient-centred care and prevention, which requires addressing the social, behavioural, and structural barriers that hinder effective TB responses particularly among underserved and high-risk groups such as men (Chikovore et al., 2020). By identifying the nature, scope, and effectiveness of gender-transformative and health promotion interventions that address the gendered dimensions of TB care, this review will generate critical evidence to guide gender-

responsive programming for men in South Africa. Furthermore, the review will inform context-specific, equity-driven approaches that enhance men's engagement in the TB care cascade contributing to the overarching goal of reducing TB incidence and mortality by 90% by 2035. In doing so, the review supports the strategic vision of eliminating TB as a public health threat through inclusive, targeted, and sustainable interventions.

Through addressing the central and sub-research questions of this scoping review, we will critically examine how concepts such as gender-transformative frameworks and health promotion theories inform the design, delivery, and implementation of interventions aimed at linking and retaining men (18 years and above) with TB in care. This reflection will enable us to assess the extent to which current interventions are grounded in sound theory, align with gender equity goals, and respond to the practical realities of health service delivery across various platforms, including community, facility, digital, and peer-led models. By mapping these conceptual and design linkages, we aim to identify critical implementation gaps and opportunities for strengthening gender-responsive TB programming in sub-Saharan Africa.

We expect that the mapped evidence will enable the identification of various strategies and interventions for linking and retaining men in TB care in the SSA region. Understanding the effectiveness of these strategies and interventions, along with the challenges in implementing them, will inform future adaptations and executions of such interventions to improve linkage and retention of men diagnosed with TB in care. The findings of the proposed scoping review will also support health system strengthening and identify gaps in intervention research involving men diagnosed with TB in SSA.

List of Abbreviations

SAA	Sub-Saharan Africa
TB	Tuberculosis
MMAT	Mixed Methods Appraisal Tool
WHO	World Health Organization

PRISMA	Preferred Reporting Items for Systematic reviews and Meta-Analyses
PCC	Population, Content and Context
BREC	Biomedical Research Ethics Committee
SDG	Sustainable Development Goals
UNAIDS	United Nations Programme on HIV/AIDS.
HIV	Human immunodeficiency virus
AIDS	Acquired immunodeficiency syndrome

Declarations

Consent for Publication- Not Applicable

Availability of the data materials- Not applicable

Competing Interests-

None declared.

Funding

Authors Contribution

SK, YS and NK led the conceptualization and design of this scoping ~~review~~. SK, YS and NK each made substantial contributions to the drafting and critical revision of the work and all authors approved the final manuscript.

Acknowledgements-

Our gratitude goes to the School of Nursing and Public Health, Discipline of Public Health, University of KwaZulu-Natal for guidance and support in developing this scoping review protocol.

Supporting Information

S 1 File PUB MED search strategy

S2 PRISMA FLOW checklist

S 3 MIXED METHOD APPRAISAL tool

S 4 DATA CHARTING form

Definition of Terms

Tuberculosis	An infectious bacterial disease caused by Mycobacterium tuberculosis, which most commonly affects the lungs. It is transmitted from person to person through the air. The symptoms of active TB include cough, chest pains, weakness, weight loss, fever and night sweats.
Gender	Refers to the characteristics of women, men, girls and boys that are socially constructed. This includes norms, behaviours and roles associated with being a woman, man, girl or boy, as well as relationships with each other. As a social construct, gender varies from society to society and can change over time.
Health	State of complete physical, mental, and social well-being, not merely the absence of disease or infirmity.
Health promotion	The process of enabling people, individually and collectively, to increase control over the determinants of health and thereby improve their health
Linkage to care	The first step in engaging in TB care and is typically defined as the completion of a first medical clinic visit within 5 to 15 days after TB diagnosis
Retention in care	Retention in care is defined as a patient's regular engagement with medical care at a health care facility after initial entry into TB clinical care.

.

References

1. Arksey, H., & O'Malley, L. (2005). Scoping studies: Towards a methodological framework. *International Journal of Social Research Methodology*, 8(1), 19–32. https://doi.org/10.1080/1364557032000119616
2. Centers for Disease Control and Prevention (CDC). (2025). *Global tuberculosis report 2025: Key facts*. Atlanta, GA: CDC.
3. Chikovore, J., Daniels, J., & Medina-Marino, A. (2024). Masculinity, health system access, and tuberculosis care engagement in South Africa. *Journal of Global Health Reports*, (in press).
4. Chikovore, J., Hart, G., Kumwenda, M., Chipungu, G. A., Desmond, N., & Corbett, E. L. (2020). Control, struggle, and emergent masculinities: A qualitative study of TB and masculinity in 56 countries. *BMC Public Health*, 20(1), 1–12. https://doi.org/10.1186/s12889-020-08895-9
5. Colvin, C. J. (2019). Strategies for engaging men in health care in South Africa: A review of best practices. *Global Health Action*, 12(1), 1581459. https://doi.org/10.1080/16549716.2019.1581459
6. Cornell, M., McIntyre, J., & Myer, L. (2015). Men and antiretroviral therapy in Africa: Our blind spot. *Tropical Medicine & International Health*, 16(7), 828–829. https://doi.org/10.1111/j.1365-3156.2011.02767.x
7. Daniels, J., Chikovore, J., & Medina-Marino, A. (2021). Masculinity, TB stigma, and access to care: A qualitative study in KwaZulu-Natal. *BMJ Global Health*, 6(2), e004436. https://doi.org/10.1136/bmjgh-2020-004436
8. Dhlakama, H., Lougue, S., Mwambi, H.G. and Ogunsakin, R.E., 2022. A Multilevel Analysis of the Associated and Determining Factors of TB among Adults in South Africa: Results from National Income Dynamics Surveys 2008 to 2017. International Journal of Environmental Research and Public Health, 19(17), p.10611.
9. Fleming, P. J., DiClemente, R. J., & Barrington, C. (2016). Masculinity and HIV: Dimensions of masculine norms that contribute to men's HIV-related sexual behaviors. *AIDS and Behavior*, 20(4), 788–798. https://doi.org/10.1007/s10461-015-1101-4

10. Gallo, J. J., Wenger, G. C., & Rodriguez, R. M. (2023). Mixed Methods Appraisal Tool (MMAT) Version 2018: Validity and reliability update. *Journal of Mixed Methods Research*, 17(2), 123–137.
11. Gibbs, A., Washington, L., & Willan, S. (2019). Gender-transformative interventions: Creating spaces for men and boys to become champions of change in South Africa. *Men and Masculinities*, 22(1), 71–90.
12. Goswami, N., 2024. A dual burden dilemma: Navigating the global impact of communicable and non-communicable diseases and the way forward. International Journal of Medical Research, 12(3), pp.65-77.
13. Hay, K., McDougal, L., Percival, V., et al. (2019). Disrupting gender norms in health systems: Making the case for change. *The Lancet*, 393(10190), 2535–2549. https://doi.org/10.1016/S0140-6736(19)30648-8
14. Jeremiah Chikovore, Madhukar Pai, Katherine Chisholm Horton, Amrita Daftary, Moses Kelly Kumwenda, Graham Hart, Elizabeth Lucy Corbett - Missing men with tuberculosis: the need to address structural influences and implement targeted and multidimensional interventions: BMJ Global Health 2020;5:e002255.
15. Koneswarakantha, B., Seebacher, D., & Durand, G. (2020). Ensuring data quality in evidence synthesis: Tools and considerations. *Systematic Reviews*, 9, 271.
16. Kuupiel, D., Bawontuo, V., & Mashamba-Thompson, T. P. (2019). Improving access to TB diagnosis in rural and remote areas in Africa: A systematic review of diagnostic strategies. *International Journal of Infectious Diseases*, 80, 20–31.
17. Levac, D., Colquhoun, H., & O'Brien, K. K. (2010). Scoping studies: Advancing the methodology. *Implementation Science*, 5, 69. https://doi.org/10.1186/1748-5908-5-69
18. Likoti, F., Sikweyiya, Y., & Kave, S. (2023). Masculinity and loss to follow-up in TB care: A critical analysis from Lesotho and South Africa. *Public Health in Practice*, 4(2), 100261.
19. MacPherson, P., Houben, R. M. G. J., Glynn, J. R., et al. (2015). Pre-treatment loss to follow-up in tuberculosis patients: A systematic review and meta-analysis. *The Lancet Infectious Diseases*, 14(7), 632–642.

20. Majid, U., Gagliardi, A. R., & Rawal, R. (2018). Conceptualizing gender-transformative health interventions: A scoping review. *PLOS ONE*, 13(11), e0209106.
21. Medina-Marino, A. (2025). Gender-blind TB care in Africa: Structural inequalities and health system design. *The Lancet Global Health*, (forthcoming).
22. Monteiro, J. F. G., de Queiroz, T. A., & Carvalho, R. D. M. (2024). Charting the data in mixed-method reviews: Lessons from TB care engagement research. *Research Synthesis Methods*, 15(2), 98–107.
23. Montgomery ND, Liomba NG, Kampani C, Krysiak R, Stanley CC, Tomoka T, et al. Accurate real-time diagnosis of lymphoproliferative disorders in Malawi through clinicopathologic teleconferences: a model for pathology services in Sub-Saharan Africa. Am J Clin Pathol. (2016) 146:423–30. doi: 10.1093/ajcp/aqw118
24. Morrison, J., Tamang, S., Mesko, N., et al. (2011). Women's health and gender equity: The role of quality assurance in research. *Health Policy and Planning*, 26(5), 347–355.
25. Mosiwa, B. (2025). Masculinity and health-seeking behaviour among men with TB in Malawi. *Global Health Review*, (in press).
26. Naidoo, P., Theron, G., Rangaka, M. X., et al. (2017). The South African National TB Survey: Delays in seeking TB care. *PLOS ONE*, 12(7), e0181031.
27. National Department of Health (NDoH), South Africa. (2021). The First National TB Prevalence Survey South Africa 2018. Pretoria: National Department of Health. Available from: https://www.knowledgehub.org.za
28. Page, M. J., McKenzie, J. E., Bossuyt, P. M., et al. (2021). The PRISMA 2020 statement: An updated guideline for reporting systematic reviews. *BMJ*, 372, n71. https://doi.org/10.1136/bmj.n71
29. Peters, M. D. J., Godfrey, C. M., McInerney, P., et al. (2020). Scoping reviews. In E. Aromataris & Z. Munn (Eds.), *JBI Manual for Evidence Synthesis*. Joanna Briggs Institute. https://doi.org/10.46658/JBIMES-20-01
30. Pollock, A., Campbell, P., Struthers, C., et al. (2023). The use of PCC framework in scoping reviews: Lessons from health equity research. *International Journal of Social Research Methodology*, 26(3), 273–285.

31. Prasanna, A., Valliappan, R., & Kumar, V. (2024). Quality assurance in evidence reviews: Best practices and challenges. *Journal of Evidence-Based Medicine*, 17(1), 12–20.
32. Rine, S., Lara, S.T., Bikomeye, J.C., Beltrán-Ponce, S., Kibudde, S., Niyonzima, N., Lawal, O.O., Mulamira, P. and Beyer, K.M., 2023. The impact of the COVID-19 pandemic on cancer care including innovations implemented in Sub-Saharan Africa: A systematic review. Journal of Global Health, 13, p.06048.
33. Sharp, A., Kave, S., & Colvin, C. (2025). Masculinities, stigma, and the failure of TB interventions in SSA. *Social Science & Medicine*, (forthcoming).
34. Sikweyiya, Y., Gibbs, A., & Medina-Marino, A. (2025). Designing gender-transformative TB interventions in Africa. *Health Promotion International* (forthcoming).
35. UNAIDS. (2018). *Blind spot: Reaching out to men and boys—Addressing a blind spot in the response to HIV*. Geneva: UNAIDS.
36. Waghule, T., Aithal, S., & Misra, S. (2021). Research protocols and methodological transparency in scoping reviews. *Journal of Research Practice*, 17(1), Article R1.
37. World Health Organization (WHO). (1986). *Ottawa Charter for Health Promotion*. Geneva: WHO.
38. World Health Organization (WHO). (2015). *The End TB Strategy: Global strategy and targets for tuberculosis prevention, care and control after 2015*. Geneva: WHO.
39. World Health Organization (WHO). (2019). *Engaging men in TB response: Strategic guidance for WHO regions*. Geneva: WHO.
40. World Health Organization (WHO). (2021). *Global tuberculosis report 2021*. Geneva: WHO.
41. World Health Organization (WHO). (2022). *Global tuberculosis report 2022*. Geneva: WHO.
42. World Health Organization (WHO). (2023). *Tuberculosis profile: WHO African Region*. Geneva: WHO.
43. World Health Organization (WHO). (2024). *Global tuberculosis report 2024*. Geneva: WHO.

Click here to access/download
Supporting Information
Pubmed search stratergy table.pdf

Click here to access/download
Supporting Information
MMAT_2018_criteria_WORD_2018-08-08.pdf

Click here to access/download
Supporting Information
EXAMPLE OF A DATA EXTRACTION TOOL USED FOR SCOPING REVIEW 2.pdf

Click here to access/download
Supporting Information
PRISMA-P-checklist Updated (1).docx
