# Supplementary material for: Gender-transformative health promotion interventions for linking and retaining tuberculosis-diagnosed adult men in care in sub-Saharan Africa: A scoping review protocol
Source: PLoS One. 2026 Jan 8;21(1):e0339666. doi: 10.1371/journal.pone.0339666 (PMC12782366; doi:10.1371/journal.pone.0339666)
Supplement: Attachment — Submitted filename: Response to reviewers.docx [file pone.0339666.s010.docx]

Dear Mickael Essouma and PLOS ONE team

We would like to sincerely thank you and the reviewers for the thoughtful and constructive feedback on our manuscript entitled manuscript title Gender-transformative health promotion interventions for linking and retaining tuberculosis-diagnosed adult men (18 years and above) in care in sub-Saharan Africa: A scoping review protocol, (Manuscript ID: PONE-D-25-36889). We greatly appreciate the time and effort invested in reviewing our work, and we believe that the comments have significantly strengthened the manuscript.

We have carefully considered all suggestions and have revised the manuscript accordingly. In the sections below, we provide a point-by-point response to each reviewer comment. All changes in the manuscript are clearly indicated in tracked changes, and we have highlighted the revisions where appropriate.

We hope that the revisions satisfactorily address the reviewers’ concerns and that the manuscript is now suitable for publication in PLOS ONE.

Sincerely,

Siyabonga Kave

On behalf of all co-authors

# **Query response sheet**

| **No.** | **Query** | **Response to Query** | **Original Wording (if applicable)** |
| --- | --- | --- | --- |
| 1 | Consider providing a reference number corresponding specifically to this scoping review | We thank the reviewers for highlighting this issue. We acknowledge that including the DOI number was an error, as we intended to provide only the OSF license number (CC0 1.0 Universal) for the scoping review protocol. The DOI number has now been removed from the document. | https://doi.org/10.17605/OSF.IO/RNF2T |
| 2 | How much confident are that these keywords will help maximize the impact and visibility of this article once published? See <https://doi.org/10.1098/rsbp.2024.1222> | We thank the reviewers for highlighting this important point to enhance the visibility of the article. We have reviewed and reorganized the list of keywords to ensure it comprehensively reflects the topic, context, methodology, public health focus, and intended outcomes. The revised keywords are: Tuberculosis, men’s health, masculinity, gender-transformative interventions, health promotion interventions, behavioural interventions, scoping review protocol, health equity, End TB Strategy 2035. | Tuberculosis, gender-transformative interventions, men’s health, linkage to care,  retention in care, health promotion, sub-Saharan Africa, scoping review. |
| 3 | The table below the key words is not warranted. These details belong to the discussion section and not in a table. | We appreciate the reviewers’ guidance regarding the table. As recommended, we have removed the table, consolidated the information, and integrated it into the discussion section. |  |
| 4 | I suggest revising this text to shorten it with brief, compelling information about the sex and age standardized incidence and prevalence of tuberculosis at the global level, in so-called LMICs, and then in sub-Saharan Africa; emphasizing TB predominance in males.  I believe some of these references might prove useful:  IJTLD OPEN 1(3):111–123 STATE OF THE ART http://dx.doi.org/10.5588/ijtldopen.23.0619  DOI:10.1371/journal.pmed.1002119  Lancet 1999; 353: 1000–01  https://doi.org/10.1016/j.pulmoe.2018.03.004  Lancet Infect Dis 2024; 24: 698–725  Foo CD et al. (2022) PLoS Med 19(1): e1003899. https://doi.org/10.1371/journal.pmed.1003899  Lee et al. BMC Public Health <https://doi.org/10.1186/s12889-022-12879-6> | We appreciate the reviewers’ suggestion aimed at strengthening the background of the topic. In response, we have revised the first two paragraphs of the article’s background as follows:  Tuberculosis (TB) remains one of the world’s deadliest infectious diseases, causing an estimated 1.25 million deaths annually (CDC, 2025) and ranking just below HIV/AIDS in global mortality (WHO, 2021). Despite major advances in diagnosis and treatment, TB disproportionately affects low- and middle-income countries (LMICs), particularly in sub-Saharan Africa (SSA) (Rine et al., 2023; Montgomery et al., 2016).  Globally, TB incidence and mortality are consistently higher among men than women. In 2021, six million men compared to 3.4 million women developed TB, with the African Region reporting 365,000 TB deaths among HIV negative individuals, men accounting for 54% (WHO, 2022; WHO, 2023). In South Africa, pulmonary TB prevalence among men aged ≥15 years was 1.6 times higher than in women (NDoH, 2021; Dhlakama et al., 2022), while a multi-country analysis across 56 nations found a male-to-female TB incidence ratio of 1.8 (Chikovore et al., 2020). These disparities highlight the urgent need to address the gendered dimensions of TB risk, care engagement, and treatment retention. | Tuberculosis (TB) remains one of the world’s deadliest infectious diseases, causing an estimated 1.25 million deaths annually (CDC, 2025) and ranking just below HIV/AIDS in global mortality burden (WHO, 2021). Despite advances in diagnosis and treatment, TB continues to disproportionately affect low-and-middle-income countries (LMICs), particularly those in sub-Saharan Africa (SSA) (Rine et al., 2023; Montgomery et al., 2016). In 2021, the African Region recorded 365,000 TB deaths among HIV-negative individuals, with men accounting for 54% and women 32% (WHO, 2023). These figures reflect a stark gender disparity in TB outcomes, driven in part by gender and social norms, and construction of masculinities that encourage men’s health-seeking avoidance behaviours, reduce diagnosis rates, and undermine treatment retention among men (Hay et al., 2019). Globally, it has been shown that TB affects more men than women. In 2021, six million men and 3.4 million women developed TB (WHO, 2022). Like other LMICs, South Africa faces a quadruple burden of diseases, which include non-communicable diseases, maternal and child health, violence and injury and HIV and TB (Goswami,2024). In South Africa, the 2018 National TB Prevalence Survey found that pulmonary TB rates among men aged 15 and older were 1.6 times higher than among women (NDoH, 2021; Dhlakama et al., 2022). A multi-country study across 56 nations reported a male-to-female TB incidence ratio of 1.8 (Chikovore et al., 2020). |
| 5 | You could revise this part of the introduction by commenting on driving factors of TB predominance in males:  Biological (genetic and hormonal?)  Psychosocial (e.g., imprisonment, homelessness, alcohol abuse, drug addiction, smoking, HIV infection)  Health behavior (e.g., reluctance to seek care due to social roles and norms)  System-related factors  These articles may be useful:  PMID: 35842266 PMCID: PMC9344469 DOI: 10.1016/j.it.2022.06.004  IJTLD OPEN 1(3):111–123 STATE OF THE ART <http://dx.doi.org/10.5588/ijtldopen.23.0619>  <https://doi.org/10.1186/s12889-024-20664-w> | We sincerely thank the reviewers for their feedback. We have carefully reviewed the passage and made the following revisions.  Research indicates that gender disparities in TB outcomes stem not only from biological differences but also from a complex interplay of structural, psychosocial, behavioral, and health system factors (Cornell et al., 2015; Colvin, 2019). Biologically, genetic and hormonal differences may influence immune responses and susceptibility to TB (DOI: 10.1016/j.it.2022.06.004). Psychosocial factors such as imprisonment, homelessness, alcohol and substance use, smoking, and HIV co-infection further increase men’s risk (IJTLD OPEN 1(3):111–123). In terms of health behavior, dominant masculine norms often discourage men from expressing vulnerability or seeking care, resulting in delayed diagnosis, poor treatment initiation, and high loss-to-follow-up rates (MacPherson et al., 2015; Naidoo et al., 2017). System-related barriers, including healthcare environments perceived as feminized, unwelcoming staff attitudes, and clinic hours incompatible with work schedules, further limit men’s engagement in TB care (Fleming et al., 2016; Daniels et al., 2021; Chikovore et al., 2024). Consequently, men face higher risks of TB complications and mortality despite the proven effectiveness of early diagnosis and treatment (WHO, 2024; Likoti et al., 2023; UNAIDS, 2018). | Scholars have argued that these disparities are shaped not only by biological differences but also by structural and social determinants, including harmful gender norms and masculine identities that discourage men from engaging with health services (Cornell et al., 2015; Colvin, 2019). Moreover, studies have shown that men are less likely to seek TB care promptly, initiate treatment early, or complete it, leading to high rates of loss to follow-up and elevated mortality (MacPherson et al., 2015; Naidoo et al., 2017). Although TB treatment is highly effective when accessed early and completed (WHO2024), men continue to face greater risks of complications and TB-related death (Likoti 4 et al., 2023; UNAIDS, 2018). In KwaZulu-Natal, South Africa, men experienced public healthcare facilities as feminized spaces, staff as unwelcoming, and that service hours were incompatible with their work and job-seeking schedules—barriers that discouraged their attendance at healthcare facilities and TB treatment adherence,(Fleming et al., 2016; Daniels et al., 2021; Chikovore et al., 2024). |
| 6 | The rest of the introduction section should focus on current knowledge and gaps in knowledge about health promotion interventions to link and retain men with TB in care pathways, thus providing the rationale for conducting the planned scoping review (see <https://doi.org/10.1186/s12874-018-0611-x>). | We sincerely thank the reviewers for this invaluable guidance. We have revised this passage as follows:  Despite the WHO’s End TB Strategy and other global frameworks emphasizing the importance of gender-responsive programming, TB interventions in sub-Saharan Africa (SSA) often remain gender-blind or gender-neutral, neglecting the role of masculinity in shaping health-seeking behaviors and care engagement (Medina-Marino, 2025; Sikweyiya et al., 2025). Although many African countries achieved TB-related Millennium Development Goals (WHO, 2021; WHO, 2023), persistent gaps in the TB care cascade remain, particularly among men, where socially entrenched ideals of stoicism and self-reliance discourage timely diagnosis, treatment initiation, and adherence (Daniels et al., 2021; Mosiwa, 2025).  Emerging evidence suggests that gender-transformative interventions—which challenge harmful masculine norms, foster supportive health systems, and promote positive health behaviors—offer a promising approach to improving TB outcomes among men (Majid et al., 2018; Colvin, 2019). When combined with health promotion interventions aimed at reducing stigma, increasing access, and sustaining engagement in care, these strategies can enhance men’s participation across the TB care continuum (WHO, 2019; WHO, 2024). Furthermore, studies highlight that co-developing interventions with men enhances cultural relevance, acceptability, and sustainability (Gibbs et al., 2019; WHO, 2024).  However, much of the available evidence comes from high-income settings, with limited synthesized knowledge on the scope, design, and effectiveness of gender-transformative and health promotion interventions for men with TB in SSA (Sharp et al., 2025). Progress in TB diagnostics and surveillance has not been matched by equivalent investments in gender-sensitive programming, especially in contexts where hegemonic masculinity norms impede care-seeking (WHO, 2023; CDC, 2025).  Given these gaps, a scoping review is warranted to map existing interventions targeting linkage to and retention in TB care for men in SSA, assess their design and delivery, and identify opportunities for strengthening gender-transformative approaches. The findings will inform the development of context-specific, equitable strategies aligned with the WHO End TB Strategy and Sustainable Development Goal 3 to end the TB epidemic by 2035 (United Nations, 2015; WHO, 2024). | These findings highlight the importance of addressing gendered health system barriers in TB control strategies. Although the WHO’s End TB Strategy and other global frameworks recognize the need for gender-responsive TB programming, many TB interventions in SSA remain gender-blind or gender-neutral, failing to address the influence of masculinity on care engagement (Medina-Marino, 2025; Sikweyiya et al., 2025). While most African countries met TB-related Millennium Development Goals (WHO, 2021; WHO, 2023), persistent gaps in the care cascade remain, particularly among men. Socially entrenched ideals that promote stoicism, discourage vulnerability, and limit help-seeking continue to impede timely diagnosis and treatment (Daniels et al., 2021; Mosiwa, 2025). Gender-transformative interventions defined as strategies that seek to challenge harmful masculine norms, promote positive health behaviours, and foster male friendly health systems offer a promising avenue for improving TB outcomes among men (Majid et al., 2018; Colvin, 2019). When integrated with health promotion interventions, such approaches can support men to construct health-promoting masculinities, while also empowering men to take control of their health (WHO,2019). The WHO (1986) defines health promotion as “the process of enabling people to increase control over, and to improve, their health.” Health promotion efforts extend beyond individual behaviour change to include the creation of supportive environments that reduce stigma, facilitate access, and promote sustained engagement in care (WHO, 2024). Emerging evidence shows that co-developing interventions with men enhances their cultural relevance, acceptability, and long-term sustainability (Gibbs et al., 2019; WHO, 2024). Despite this growing evidence largely from the Global North, there 5 remains limited synthesized evidence on the scope, design, and impact of gender transformative and health promotion interventions targeting men with TB in SSA. Furthermore, while progress has been made in TB diagnostics and surveillance in high-burden countries such as South Africa, Nigeria, and Zambia (WHO,2023; CDC, 2025), the implementation of gender-sensitive approaches remains weak, particularly in settings where masculinity undermines care-seeking (Sharp et al., 2025). Emerging evidence indicates that co-developing interventions with men enhances their cultural relevance, acceptability, and long-term sustainability (Gibbs et al., 2019; WHO, 2024). However, much of this evidence originates from the Global North, with limited synthesized evidence available on the scope, design, and impact of gender transformative and health promotion interventions targeting men diagnosed with TB in sub-Saharan Africa (SSA). While notable progress has been made in TB diagnostics and surveillance (CDC, 2025), the implementation of gender-sensitive and gender-transformative approaches remains weak, especially in settings where hegemonic masculinity norms undermine men’s engagement in care (Sharp et al., 2025). This gap is particularly concerning in SSA, where TB incidence and mortality remain high among men. Ending the global TB epidemic is a core component of Sustainable Development Goal 3 (SDG 3), which seeks to "ensure healthy lives and promote well-being for all at all ages." Specifically, Target 3.3 aims to end the epidemics of AIDS, TB, malaria, and neglected tropical diseases by 2030 (United Nations, 2015). Achieving this target requires not only biomedical advancements but also inclusive, context-specific strategies that address the social and structural determinants of TB, including gender based disparities in access, treatment, and outcomes (Banda, 2024; Haji, 2024). Given the urgency of ending TB by 2035 in line with the WHO’s End TB Strategy (WHO, 2024), there is a critical need to examine the landscape of gender transformative and health promotion strategies tailored specifically for men in SSA. A scoping review is therefore warranted to map existing interventions, explore how they are designed and delivered, evaluate their effectiveness, and identify implementation |
| 7 | This statement belongs to the introduction section where the authors should be stating the aims of the scoping review for which this protocol is being prepared. | We thank the reviewers for their valuable guidance. In response, we have moved the statement to the introductory section of the manuscript to enhance clarity and coherence." |  |
| 8 | Consider specifying the theme. | We thank the reviewers for their valuable guidance. In response, we have made the following change in the manuscript: This scoping review forms part of a PhD study titled Developing a Gender-Transformative Health Promotion Intervention for Men Diagnosed with TB in the Eastern Cape Province, South Africa. |  |
| 9 | Consider providing the IRB approval letter as supplemental material to this manuscript. | We thank the reviewers once again for their guidance. In line with their recommendation, we have attached the Ethics Approval Letter as a supplementary document to this submission. |  |
| 10 | Please, avoid repeating the aim of the scoping review throughout the manuscript. | We thank the reviewers for their valuable guidance. In response, we have removed the repeated aim statement throughout the manuscript and integrated it solely within the introductory section as advised. |  |
| 11 | Did you wish to state: "What evidence exists on gender transformative health promotion interventions aimed at strengthening the linkage to and retention in care of adult men diagnosed with TB in sub-Saharan Africa?" | We thank the reviewers for pointing this out. The error has been corrected as advised, and the statement now accurately reflects the intended wording. |  |
| 12 | It seems to me that this is still the central research question. Consider removing. | We thank the reviewers for their guidance. In response, we have removed the first sub-question as recommended |  |
| 13 | Is it possible to clarify the secondary research questions as:  What are barriers and facilitators of gender-transformative health promotion interventions aimed at strengthening linkage to and retention in care?  What are the methods/means and administration platforms of these interventions?  What are the outcomes of these interventions? | We sincerely thank the reviewers for their valuable guidance. In response to their recommendations, we have revised the secondary research questions in the manuscript as follows:   1. What are the barriers to and facilitators of gender-transformative health promotion interventions aimed at strengthening linkage to and retention in care? 2. What methods, strategies, and delivery platforms are used to implement these interventions? 3. What are the outcomes of these interventions? |  |
| 14 | To my knowledge there is not such a sub-section in the materials and methods section based on Arksey and O'Malley’s methodology. Furthermore, it seems to me that in this subsection you were presenting the eligibility criteria which you have repeated below. Consider presenting eligibility criteria under Step 3 Study Selection, as seen in this article: https://doi.org/10.1371/journal.pone.031491. I agree with the framing of the eligibility criteria based on the JBI derived PCC concept for scoping reviews in a table (eg as seen here: https://doi.org/10.1371/journal.pone.0322753 on in the text. However, you would like to inform readers that you framed the eligibility criteria based on the JBI PCC concept for scoping reviews before specifying those criteria. With regards to the population to include, consider providing information about the the included age group, sex, and socio-economic characteristics such as ethnicity, race and income. For the concept consider providing information about three secondary research questions proposed above. Regarding the context consider providing the information about the continents, countries and localities (urban vs rural) where the studies were conducted as well as the language included in publications. | We would like to thank the reviewer for this valuable guidance. We have revised the manuscript accordingly by framing the eligibility criteria based on the JBI-derived PCC concept for scoping reviews and have moved this section under Stage 3: Study Selection, as recommended.  In the revised version, we clearly inform readers that the eligibility criteria are derived from the PCC framework before specifying the criteria. We have also incorporated additional details regarding the Population (age group, sex, and socio-economic characteristics such as ethnicity, race, and income), the Concept (including the three secondary research questions proposed), and the Context (continents, countries, localities (urban vs rural) and the language of included publications. |  |
| 15 | Could you reframe these two sentences like this: Relevant literature on men diagnosed with TB will be searched through electronic databases such as PubMed via (MEDLINE), Web of Science, PsychInfo, Google Scholar World Health Organization library database. Could you also follow that sentence with a brief explanation about why you chose those databases as did these authors: https://doi.org/10.1371/journal.pone.0322753? | We would like to thank the reviewers for their helpful suggestions regarding the selection of databases and the rationale for their inclusion. The provided guidance has enabled us to refine our methodology and ensure a comprehensive and systematic search strategy. In response here bolow is a revised passage:  Relevant literature on men diagnosed with TB will be searched through electronic databases, including PubMed via MEDLINE, Cochrane Library, Embase.com, Global Health, Web of Science, PsycINFO, Google Scholar, Africa-Wide Information, and the World Health Organization (WHO) library database.  These databases were strategically selected for their relevance to the scope of this review, which focuses on gender-transformative health promotion interventions for men diagnosed with tuberculosis (TB) in sub-Saharan Africa. MEDLINE, Embase, Global Health, and the Cochrane Library were included due to their comprehensive coverage of biomedical and public health research, ensuring that clinical, epidemiological, and intervention studies relevant to TB management and health promotion were captured. |  |
| 16 | Can you complete the sentence with names of the authors who will be involved this search step? | We thank the reviewers for providing this valuable guidance. We added the initials of the three reviewers in the corrected version of the manuscript. In response the sentence has been revised as follows:  The principal investigator (SK), together with the primary supervisor YS, and the co-supervisor, NK will ensure the validity of the search strategy to be used. |  |
| 17 | See my comments about the eligibility criteria above. | We would like to thank the reviewers once again for their valuable input. In response to the comments regarding the eligibility criteria, we have revised the PCC table and moved it to Stage 3 of the manuscript. The revised table now clearly specifies the Population (including age group, sex, and socio-economic characteristics such as ethnicity, race, and income), the Concept (addressing the three secondary research questions), and the Context (including continents, countries, localities (urban vs rural), and publication language). These revisions ensure that the eligibility criteria are fully aligned with the JBI-derived PCC framework and provide a structured approach for selecting studies relevant to our scoping review |  |
| 18 | This sentence belongs to the study selection sub-section below:  In addition, the results of the study will be exported to EndNote library for deduplication and organizing | We sincerely thank the reviewers for this important guidance. In response, the sentence has been moved to the Study Selection subsection as recommended. |  |
| 19 | Consider revising this sentence clarifying the key search concepts and terms. Consult expert librarians. | We thank the reviewers for their valuable feedback regarding the clarity of the search concepts and terms. In response, we have revised the search strategy to clearly reflect the key concepts of tuberculosis, men’s health and masculinity, gender-transformative and behavioral interventions, health promotion, health equity, and the End TB Strategy 2035. Expert librarians were consulted to ensure the search strings included appropriate controlled vocabulary (e.g., MeSH, Emtree) as well as free-text terms for optimal sensitivity and specificity across databases. The revised search strategy, now fully documented in the supplementary file, provides a transparent and reproducible approach aligned with best practices for scoping reviews. |  |
| 20 | Not warranted:  Studies that do not meet the eligibility criteria will be excluded | We appreciate the reviewer’s observation. We acknowledge that the statement “Studies that do not meet the eligibility criteria will be excluded” may be redundant. To address this, we have integrated the exclusion criteria directly into the Study Selection subsection under Stage 3, alongside the inclusion criteria, to improve clarity and avoid repetition |  |
| 21 | It is important to note that exclusion criteria are virtually criteria that lead to the exclusion of records that could be deemed eligible based on inclusion criteria, but are finally excluded based due to other variables such as lack of full text after attempting to retrieve it from the authors, duplicate or salami slice reports, articles published in languages other than those selected by the authors, articles not presenting peer reviewed original search on the studied topic eg reviewers, editorials. So in my opinion the exclusion criteria presented here should be replaced by those ones. | We appreciate the reviewers’ feedback on the inclusion and exclusion criteria. As recommended, we have revised this section to clearly distinguish between studies that meet inclusion criteria and those excluded for practical or methodological reasons. Specifically, the revised exclusion criteria now address issues such as lack of full-text availability despite attempts to retrieve it, duplicate or salami-sliced reports, publications in languages outside the review scope, and articles that do not present original peer-reviewed research (e.g., editorials, opinion pieces, or narrative reviews).  These revisions ensure greater clarity, align with best practices for scoping reviews, and improve the methodological rigor of the protocol. |  |
| 22 | The PRISMA flow chart is not relevant for a scoping review protocol. Suggest deleting it. | We thank the reviewers for this helpful observation. In line with the advice provided, we have removed the PRISMA flow diagram from the manuscript to ensure the protocol aligns with the appropriate standards for scoping reviews. |  |
| 23 | The QA subsection is not necessary, but the information provided here could be moved to the description of data charting. | We sincerely thank the reviewers for their valuable feedback. As per the advice provided, we have carefully revised the manuscript and moved the information previously included under the Quality Assurance subsection to the Charting the Data section to ensure better alignment and clarity. |  |
| 24 | Consider classifying data into categories as seen in: https://doi.org/10.1371/journal.pone.0322753 | We sincerely thank the reviewers for their valuable guidance. In response, we have revised the data charting form as advised, and the updated version has been included as Fig S4 for reference. |  |
| 25 | This section needs to be shortened, see how authors of previously published scoping review protocol articles provided the above framed this subsection. | We thank the reviewers for this valuable recommendation. In response, we have revised and shortened the subsection on data synthesis, reporting, consultation, data sharing, and timeline to align with the style and conciseness commonly seen in published scoping review protocols, while retaining all essential methodological details. |  |
| 26 | coping review | Thank you for pointing this out. The use of “coping review” was an inadvertent typographical error. The correct term is “scoping review,” which has been corrected throughout the manuscript to ensure consistency and accuracy. |  |
| 27 | Revise the timeline as we are already past August 2025. | We thank the reviewers for their guidance. In response, we have updated the scoping review timelines. The initial search will be conducted on October 31, 2025. Title and abstract screening, along with full-text screening, will take place from November 2025 to January 2026. Data extraction is expected to be completed by March 2026, followed by analysis and synthesis of results from March to May 2026. We anticipate finalizing the results and submitting the manuscript for publication by June 2026 |  |
| 28 | Before getting into the subsection (Discussion), consider the following sub-sections at the end of the materials and methods section as seen in the previously mentioned published scoping review protocols and in another scoping review protocol articlehttps://doi.org/10.1371/journal.pone.0328645.  Data sharing, protocol deviation, patient and public involvement. Consider adding an ethics and dissemination statement just after the Discussion section as did these authors: https://doi.org/10.1371/journal.pone.0328645 | We thank the reviewers for their insightful suggestions. In response, we have added the following sub-sections at the end of the Materials and Methods section: Data Sharing, and consultation with stakeholders. Additionally, we have included an Ethics and Dissemination statement immediately following the Discussion section, in alignment with the structure used in published scoping review protocols, including the example provided <https://doi.org/10.1371/journal.pone.0328645> |  |
| 29 | The proposed scoping review will map and synthesise existing literature on gender-transformative and health promotion interventions implemented in sub-Saharan Africa (SSA) that aim to strengthen linkage to care and retention among men (18 years and above) diagnosed with TB. | Thank you for this comment. We have revised the aim of the scoping review to provide greater clarity and specificity. The aim is now framed as follows:  “A scoping review is warranted to map and synthesize existing evidence on the design, implementation, and outcomes of gender-transformative health promotion interventions aimed at improving linkage to and retention in care among adult men diagnosed with all forms of TB in sub-Saharan Africa. |  |
| 30 | Consider revising this text, avoiding repetition of information mentioned in the introduction section and focusing on the provision of expected strengthens and weaknesses of the methods proposed for the upcoming scoping review for which protocol is being prepared. | We thank the reviewers for their constructive feedback. As recommended, the discussion section of the manuscript has been revised to provide a critical reflection on the anticipated strengths and weaknesses of the proposed scoping review methods. Specifically, the revised discussion now highlights how the structured and iterative approach, diverse data sources, stakeholder engagement, and use of established synthesis frameworks will strengthen the comprehensiveness, transparency, and applicability of the findings. At the same time, it acknowledges potential limitations, including selection bias due to language restrictions, heterogeneity across interventions, and the inability to quantitatively assess intervention effectiveness—together with mitigation strategies to address these challenges.  We believe these revisions strengthen the methodological rigor and clarity of the protocol as suggested. | A core pillar of the End TB Strategy is integrated, patient-centred care and prevention, which requires addressing the social, behavioural, and structural barriers that hinder effective TB responses particularly among underserved and high-risk groups such as men (Chikovore et al., 2020). By identifying the nature, scope, and effectiveness of gender-transformative and health promotion interventions that address the gendered dimensions of TB care, this review will generate critical evidence to guide gender-responsive programming for men in South Africa. Furthermore, the review will inform context-specific, equity-driven approaches that enhance men’s engagement in the TB care cascade contributing to the overarching goal of reducing TB incidence and mortality by 90% by 2035. In doing so, the review supports the strategic vision of eliminating TB as a public health threat through inclusive, targeted, and sustainable interventions.  Through addressing the central and sub-research questions of this scoping review, we will critically examine how concepts such as gender-transformative frameworks and health promotion theories inform the design, delivery, and implementation of interventions aimed at linking and retaining men (18 years and above) with TB in care. This reflection will enable us to assess the extent to which current interventions are grounded in sound theory, align with gender equity goals, and respond to the practical realities of health service delivery across various platforms, including community, facility, digital, and peer-led models. By mapping these conceptual and design linkages, we aim to identify critical implementation gaps and opportunities for strengthening gender-responsive TB programming in sub-Saharan Africa.  We expect that the mapped evidence will enable the identification of various strategies and interventions for linking and retaining men in TB care in the SSA region. Understanding the effectiveness of these strategies and interventions, along with the challenges in implementing them, will inform future adaptations and executions of such interventions to improve linkage and retention of men diagnosed with TB in care. The findings of the proposed scoping review will also support health system strengthening and identify gaps in intervention research involving men diagnosed with TB in SSA. |
| 31 | The list of abbreviations is not needed. Spell out abbreviations when first mentioned. | We thank the reviewers for this suggestion. In response, the list of abbreviations has been removed from the protocol, and all abbreviations are now spelled out when first mentioned in the text |  |
| 32 | Consent for Publication- Not Applicable | We thank the reviewer for highlighting this. In this case, consent for publication is not applicable. This manuscript reports a scoping review of published literature and does not involve the collection of primary data from human participants. As such, no individual participant data are included, and consent for publication is therefore not required. |  |
| 33 | Consider providing full funding statement. | Thank you for this comment. We confirm that this research received no specific grant from any funding agency in the public, commercial, or not-for-profit sectors |  |
|  | Consider moving the definition of terms section to the Methods section and align definitions with recent evidence (e.g., Lancet Glob Health 2024; 12(5): e737–e739). | We thank the reviewer for this valuable suggestion. In response, we have relocated the Definition of Terms section to the Methods section to enhance methodological clarity and ensure alignment with best practices in scoping review reporting. |  |
| 34 | Strengthen and update references, reducing their number if possible. | We thank the reviewers for the suggestion to strengthen and streamline the reference list. In response, we conducted a thorough update of the references to ensure that the most current, relevant, and high-impact studies are represented. The total number of references has increased from 42 to 45 following the inclusion of recent and seminal publications that provide stronger empirical and theoretical support for the revised sections of the manuscript, particularly those addressing gender-transformative health promotion interventions and men’s engagement in TB care.  While duplicate and less essential citations were removed to improve clarity and focus, the addition of these key sources enhances the rigor, currency, and comprehensiveness of the evidence base underpinning the review. This ensures that the reference list now reflects the most authoritative and up-to-date literature aligned with the refined scope of the manuscript. |  |

**Reviewers additional comments**

| **No.** | **Query** | **Response to Query** | **Original Wording (if applicable)** |
| --- | --- | --- | --- |
| 1 | Please ensure that your manuscript meets PLOS ONE's style requirements, including those for file naming.  . | We thank the reviewer for this comment. We have ensured that the revised manuscript now fully complies with PLOS ONE’s style requirements, including adherence to all file naming conventions for text, tables, figures, and supplementary materials. |  |
| 2 | Please amend either the title on the online submission form (via Edit Submission) or the title in the manuscript so that they are identical. | We thank the reviewer for pointing this out. We have amended the title so that it is now identical on both the online submission form and in the manuscript. |  |
| 3 | Please amend either the abstract on the online submission form (via Edit Submission) or the abstract in the manuscript so that they are identical. | We thank the reviewer for highlighting this. We have amended the abstract so that it is now identical in both the online submission form and the manuscript. |  |
| 4 | If the reviewer comments include a recommendation to cite specific previously published works, please review and evaluate these publications to determine whether they are relevant and should be cited. There is no requirement to cite these works unless the editor has indicated otherwise. | We thank the reviewer for suggesting additional references. We have carefully reviewed the recommended publications and evaluated their relevance to our study. Where appropriate, we have incorporated citations to strengthen the manuscript. |  |
| 5 | The main limitation with this scoping review protocol is that the aim of the scoping review for which the protocol is being conceived is unclear. Along the same line, the authors did not clearly state in the Materials and Methods section whether the review will assess contextual and culturally-sensitive gender transformative health promotion interventions as well as the role of key actors involved in the conception and implementation of those interventions, although they mentioned the potential driving role of culture-specific factors for the predominance of tuberculosis in males and the global health community as an important audience for this research. See Lancet Glob Health 2025;13: e1627–35. | We thank the reviewer for this valuable feedback. In response, we have made the following revisions to strengthen the clarity and scope of the scoping review protocol:  Clarified the Aim:  We have revised the aim to explicitly state that the review will map and synthesize evidence on the design, implementation, contextual and cultural relevance, and outcomes of gender-transformative health promotion interventions aimed at improving linkage to and retention in care among adult men diagnosed with TB in sub-Saharan Africa.  Contextual and Culturally Sensitive Interventions:  In the Materials and Methods section, we have explicitly noted that the review will assess the contextual and culturally sensitive dimensions of identified interventions, acknowledging the critical role of cultural norms and context-specific factors in shaping men’s health-seeking behaviors and TB outcomes.  Role of Key Actors:  We have also incorporated language indicating that the review will examine the role of key actors—including healthcare providers, policymakers, program implementers, and men with lived experiences of TB—in the conception, implementation, and delivery of gender-transformative interventions.  Incorporation of Recommended Literature:  We have reviewed and cited the suggested article (Lancet Glob Health 2025;13: e1627–35) to further strengthen the background and rationale for assessing cultural and contextual factors and stakeholder roles in intervention implementation.  These revisions ensure that the aim, scope, and methodological approach are now clearly aligned with the reviewer’s recommendations and with best practices for scoping reviews. |  |
| 3 | Furthermore, it is unclear which countries they were referring to when reporting on LMICs in the manuscript. See BMJ Glob Health. 2022 Jun;7(6):e009704. doi: 10.1136/bmjgh-2022-009704 and https://doi.org/10.1007/s10741-025-10550-x. | Thank you for this observation. We acknowledge that the initial version of the manuscript did not specify the countries referred to as low- and middle-income countries (LMICs). To address this, we have now clarified in the Methods – Eligibility Criteria section that the scoping review will focus on studies conducted in sub-Saharan African countries classified as LMICs according to the World Bank income classification at the time of the review. We have also provided a supplementary table listing all eligible countries to ensure transparency and alignment with the referenced literature (BMJ Glob Health. 2022;7:e009704; https://doi.org/10.1007/s10741-025-10550-x  ). |  |
| 4 | It is important to specify throughout the manuscript whether the tuberculosis health promotion interventions you would like to assess are preventive and/or curative health promotion interventions, and whether you are addressing individual-level and/or mass/community-based interventions | We thank the reviewer for highlighting this important point. In the revised manuscript, we have clarified throughout that the scoping review will include both preventive and curative health promotion interventions that contribute to linkage to care, treatment initiation, adherence, and retention among adult men diagnosed with TB in sub-Saharan Africa.  Furthermore, we specify that the review will consider interventions implemented at both the individual level (e.g., one-on-one counseling, patient navigation, peer-support programs) and the community or mass level (e.g., community outreach, group education sessions, mass media campaigns). |  |
| 5 | Are you studying tuberculosis at large or a specific form of tuberculosis? This information should also be provided throughout the manuscript, including in Definitions sub-section will of the Materials and Methods section. See https://doi.org/10.1371/journal.pone.0322753 and Lancet Glob Health. 2024 May;12(5):e737-e739. | We thank the reviewer for this observation. In the revised manuscript, we have clarified that the scoping review will include all forms of tuberculosis (TB) among adult men, including:  Pulmonary TB (microbiologically confirmed or clinically diagnosed)  Extra-pulmonary TB  Latent TB  Active TB  Drug-sensitive and drug-resistant TB  This clarification has been incorporated throughout the manuscript, including in the Definitions subsection of the Materials and Methods, the Eligibility Criteria table, and the Concept section, to ensure readers clearly understand the scope of TB under investigation. |  |
| 6 | What is the relevance of the MMAT questionnaire in the supplemental material accompanying this submission? | Thanks to the reviewer for the highlighting this. The Mixed Methods Appraisal Tool (MMAT) 2018 included in the supplemental material is used to assess the methodological quality of the studies included in the scoping review. Although study quality will not determine inclusion, the MMAT allows us to systematically evaluate the rigor, reliability, and relevance of quantitative, qualitative, and mixed-methods studies. This ensures transparency and supports the interpretation of findings by highlighting strengths and limitations across diverse study designs. |  |
| 7 | The data extraction chart provided as a suplemental material seems to be that of a critical interpretive synthesis whereas this protocol is for a scoping review. Pay attention to this matter. | We thank the reviewer for this observation. We acknowledge that the previously provided data extraction chart was aligned with a critical interpretive synthesis rather than a scoping review. In response, we have developed a revised data extraction tool specifically tailored for a scoping review. This updated tool focuses on systematically capturing key information across studies, including study characteristics, population, intervention details, implementation factors, outcomes, and contextual considerations, without critically appraising effectiveness. The revised tool aligns with the objectives of mapping evidence on gender-transformative health promotion interventions for men diagnosed with TB in sub-Saharan Africa and is included as supplemental material in this submission. |  |
| 8 | Consider describing the Arksey and O'Malley methods for scoping reviews as did these authors (https://doi.org/10.1371/journal.pone.0314914) and integrating the comments made about review methods. | We thank the reviewer for the suggestion. The methodology section has been revised to clearly describe that this scoping review follows the framework by Arksey and O’Malley (2005), refined by Levac et al. (2010). The six-step process—including research question identification, study selection, data charting, and stakeholder consultation—has been explicitly outlined in the protocol. |  |
| 9 | Are you planning to add a «Consultation with stakeholders» sub-section in the Materials and Methods section as optional last step of the Arksey and O'Malley methods for scoping reviews? | Yes, a “Consultation with stakeholders” sub-section has been included as the final step in the Materials and Methods section, in line with Arksey and O’Malley’s framework. This step will engage healthcare workers, TB program managers, policymakers, and men with lived TB experience to ensure that the findings are contextually relevant, actionable, and inform the design of culturally appropriate, gender-transformative interventions. |  |
| 10 | Finally, consider conforming to PLOS One guidelines for formatting manuscripts. For instance, the abstract and references are not recorded as recommended by PLOS One both in the main text and in the reference section and numbering the manuscript's lines to ease its assessment. See how the abstract should appear in the manuscript: <https://doi.org/10.1371/journal.pone.0314914> | We appreciate the reviewer’s observation regarding PLOS ONE formatting requirements. We have revised the manuscript to conform to PLOS ONE guidelines by:  Reformatting the abstract to follow the journal’s recommended structured style.  Updating all in-text citations and the reference list according to PLOS ONE (Vancouver) format and ordering references sequentially. |  |
| 11 | Does the manuscript provide a valid rationale for the proposed study, with clearly identified and justified research questions?  The research question outlined is expected to address a valid academic problem or topic and contribute to the base of knowledge in the field.  Reviewer #1: No  Reviewer #2: No  Reviewer #3: Yes  Reviewer #4: Yes | We thank the reviewers for their comments regarding the rationale and research questions. We have carefully revised the Background section to provide a clear and evidence-based rationale for the proposed scoping review. In response to the feedback, we have also clarified the research questions, linking them directly to the identified gaps and specifying the population (adult men), interventions (preventive and curative gender-transformative health promotion interventions), outcomes (linkage to care, treatment initiation, adherence, retention). We believe that these revisions strengthen the rationale and justification for the scoping review and address the concerns raised by reviewers #1 and #2. |  |
| 12 | Is the protocol technically sound and planned in a manner that will lead to a meaningful outcome and allow testing the stated hypotheses?  The manuscript should describe the methods in sufficient detail to prevent undisclosed flexibility in the experimental procedure or analysis pipeline, including sufficient outcome-neutral conditions (e.g. necessary controls, absence of floor or ceiling effects) to test the proposed hypotheses and a statistical power analysis where applicable. As there may be aspects of the methodology and analysis which can only be refined once the work is undertaken, authors should outline potential assumptions and explicitly describe what aspects of the proposed analyses, if any, are exploratory.  Reviewer #1: Partly  Reviewer #2: No  Reviewer #3: Yes  Reviewer #4: Partly | We thank the reviewers for their comments. As a scoping review, the primary aim is to map and synthesize existing evidence on gender-transformative health promotion interventions for adult men with TB in sub-Saharan Africa, rather than test specific hypotheses.  The review uses a structured, transparent methodology (Arksey & O’Malley, 2005; Levac et al., 2010) and the PCC framework (Pollock et al., 2023) to ensure outcome-neutral study selection, data extraction, and synthesis. Exploratory elements include identifying barriers and facilitators, examining contextual and cultural influences, and mapping intervention strategies and outcomes.  These methods provide sufficient rigor to produce meaningful insights while maintaining transparency and minimizing bias. |  |
| 13 | Is the methodology feasible and described in sufficient detail to allow the work to be replicable?   Descriptions of methods and materials in the protocol should be reported in sufficient detail for another researcher to reproduce all experiments and analyses. The protocol should describe the appropriate controls, sample size calculations, and replication needed to ensure that the data are robust and reproducible.  Reviewer #1: No  Reviewer #2: No  Reviewer #3: Yes  Reviewer #4: Yes | We thank the reviewers for their feedback. The methodology is clearly described, including database selection, search strategies, eligibility criteria (PCC framework), data charting, and synthesis approaches, ensuring transparency and reproducibility. As this is a scoping review, formal sample size calculations and experimental controls are not applicable, but steps such as dual independent screening, piloting of the data extraction tool, and use of established quality appraisal tools (MMAT, JBI, Newcastle-Ottawa, Jadad) are included to ensure rigor and reliability. |  |
| 14 | Have the authors described where all data underlying the findings will be made available when the study is complete?  The [PLOS Data policy](https://track.editorialmanager.com/CL0/https:%2F%2Fjournals.plos.org%2Fplosone%2Fs%2Fmaterials-and-software-sharing/1/010f019904b062a7-8ce15b7d-8d1e-4125-a2f3-815e3512f411-000000/duUAAH2Nco0Tjq9tJBR3eHJ_wd2td2ndZB8qjoNKrn8=226) requires authors to make all data underlying the findings described in their manuscript fully available without restriction, with rare exception, at the time of publication. The data should be provided as part of the manuscript or its supporting information, or deposited to a public repository. For example, in addition to summary statistics, the data points behind means, medians and variance measures should be available. If there are restrictions on publicly sharing data—e.g. participant privacy or use of data from a third party—those must be specified.  Reviewer #1: Yes  Reviewer #2: Yes  Reviewer #3: Yes  Reviewer #4: No | We thank the reviewer for highlighting this point. In line with PLOS data-sharing policies, all data extraction sheets, metadata, and synthesized findings from this scoping review will be made publicly available via the Open Science Framework at the time of publication, ensuring full transparency and accessibility. Any restrictions will be clearly noted if they arise. |  |
| 15 | Is the manuscript presented in an intelligible fashion and written in standard English?  PLOS ONE does not copyedit accepted manuscripts, so the language in submitted articles must be clear, correct, and unambiguous. Any typographical or grammatical errors should be corrected at revision, so please note any specific errors here.  Reviewer #1: No  Reviewer #2: Yes  Reviewer #3: Yes  Reviewer #4: Yes  Reviewer #1  It is well known that TB Globally affects men more than women, hence is not only an SSA condition. The added value of this review over what is published is missing. | We acknowledge Reviewer #1’s comment regarding language clarity. The manuscript has been carefully revised to ensure it is written in standard, clear, and unambiguous English. All typographical and grammatical errors have been corrected to improve readability and presentation. |  |
| 16 | Reviewer #1  Until now, there are no significant biological experimental evidence supporting this gender increased risk. However, certain social conditions like imprisonment history or being homeless was not addressed here nor other comorbidities like being HIV positive, lung cancer, previous TB treatment, etc. | We thank Reviewer #1 for this observation. We have clarified in the background that, while biological evidence for male-biased TB risk is limited, social and structural determinants—including incarceration history, homelessness, HIV co-infection, prior TB treatment, and other comorbidities—significantly contribute to increased TB risk and poor care engagement among men. References to these factors have been explicitly included to contextualize gender disparities in TB outcomes |  |
| 17 | Reviewer #1:  Risk associated addictions like alcohol and drug abuse are missing also in this work. I believe that the increased risk of having TB and being male was not properly addressed in this work. | We appreciate Reviewer #1’s comment. We have now explicitly included alcohol and substance use as key psychosocial determinants that increase TB risk among men in the background section. The manuscript clarifies that the heightened TB burden among men arises from a combination of biological, behavioral, and social factors, including addictions, HIV co-infection, previous TB treatment, and structural barriers, thereby providing a more comprehensive explanation of male-associated TB risk. |  |
| 18 | Reviewer #2:  The study is about to provide a review protocol. Protocols are according to standard guidelines but to validate the protocol, study needs to be done in real-time. As authors have mentioned , the final study manuscript will be submitted by January 2026, that time it can be considered. | We appreciate the reviewer’s attention to the study timeline. The protocol includes a detailed, realistic schedule to ensure systematic completion of each stage of the scoping review. The initial search is planned for October 31, 2025, followed by title/abstract and full-text screening from November 2025 to January 2026. Data extraction is scheduled for completion by March 2026, with analysis and synthesis from March to May 2026, and final manuscript submission anticipated by June 2026. This timeline ensures that the review will be conducted systematically and completed within a feasible timeframe. |  |
| 19 | Reviewer #2:  Authors can also look into statistical analysis of results obtained in meta-analysis for significance of interventions. | We thank the reviewer for this suggestion. As this is a scoping review, the primary aim is to map and synthesize existing evidence rather than to conduct formal hypothesis testing or meta-analytic statistical comparisons. However, where sufficient quantitative data are available, we will extract key effect measures and summarize trends descriptively. Any exploratory meta-analyses or statistical assessments of intervention significance will be clearly noted as secondary or exploratory analyses in the review, ensuring transparency. |  |
| 20 | Reviewer #3:  The protocol is well written and all the necessary details are provided. When arrried out, will provide importnat inputs to TB program mangers to address the gender speciifc access to high quality TB Care. | We thank Reviewer #3 for the positive feedback and for highlighting the potential practical value of this scoping review. We agree that the findings will provide important insights for TB program managers to strengthen gender-responsive approaches and improve men’s access to high-quality TB care. |  |
| 21 | Reviewer #4:  Authors are addressing a relevant subject to current End TB Strategy | We thank Reviewer #4 for acknowledging the relevance of our study. We agree that this scoping review aligns closely with the WHO End TB Strategy and will contribute to evidence-based, gender-responsive interventions to improve TB care for men in sub-Saharan Africa. |  |
| 22 | Editor #1:  BACKGROUND  Could you provide evidence in numbers that men in SSA (or elsewhere, if no data available) were less likely to complete TB treatment, and more likely to default/fail treatment. I've come across a couple of articles from SSA and worth mentioning to strengthen the rationalisation of the study that aimed to look for interventions to improve retention in care. | Thank you for your valuable suggestions. We have addressed the points as follows:  Evidence on men’s TB treatment outcomes: We have incorporated SSA-specific data demonstrating that men are less likely than women to complete TB treatment and are more likely to experience treatment default or failure. For example, men had a 1.3–1.8 times higher risk of treatment interruption in multi-country SSA studies (Chikovore et al., 2020; Naidoo et al., 2017), and in South Africa, men accounted for 60% of TB treatment interruptions with lower overall treatment completion rates compared to women (Dhlakama et al., 2022; Likoti et al., 2023). These statistics strengthen the rationale for focusing on interventions to improve linkage, adherence, and retention among men.  Rationale for the timeframe: In response to your concern, we have extended the literature search period to cover studies published from 2000 to 2024. This allows us to capture earlier interventions that may provide lessons for contemporary gender-transformative TB programs and to identify changes in intervention design, implementation, and outcomes over time.  Inclusion of grey literature: We have retained grey literature (e.g., government reports, policy briefs, program evaluations) because such sources provide critical information on real-world implementation, contextual factors, and intervention outcomes that are often not captured in peer-reviewed publications. We have clearly justified this inclusion in the protocol to ensure a comprehensive mapping of gender-transformative interventions.  We believe these revisions address your concerns and strengthen the background and rationale of our scoping review protocol. |  |
| 23 | Not sure you have provided the rationale for choosing only the past 10 years for your study. Have authors observed improvement in interventions over this period? You might have missed good interventions before this period and weaken/narrow your findings. I would probably extend the the period of search and skip the grey materials, as not necessarily peer-reviewed, unless if you rationalise this. | Thank you for the comment. We have extended the literature search period to cover studies published from 2000 to 2024 to capture earlier interventions that may inform contemporary gender-transformative TB programs. This allows us to identify trends and lessons over time, rather than limiting the review to the past 10 years.  Regarding grey literature, we have included it (e.g., government reports, policy briefs, program evaluations) because such sources provide valuable insights into real-world implementation, contextual factors, and outcomes that are often not reported in peer-reviewed publications. This inclusion is explicitly justified in the protocol to ensure a comprehensive mapping of interventions. |  |
