# Supplementary material for: Gender-transformative health promotion interventions for linking and retaining tuberculosis-diagnosed adult men in care in sub-Saharan Africa: A scoping review protocol
Source: PLoS One. 2026 Jan 8;21(1):e0339666. doi: 10.1371/journal.pone.0339666 (PMC12782366; doi:10.1371/journal.pone.0339666)
Supplement: Attachment — Submitted filename: PONE-D-25-36889_R1_reviewed by Mickael Essouma.docx [file pone.0339666.s009.docx]

# **Gender-transformative health promotion interventions for linking and retaining tuberculosis-diagnosed adult men in care in sub-Saharan Africa: A scoping review protocol**

Authors: Siyabonga Kave^1^, Yandisa Sikweyiya^1,2,3^, Nelisiwe Khuzwayo^1^.

1. School of Nursing and Public Health, College of Health Sciences, Howard College Campus, University of KwaZulu-Natal, Durban, South Africa

2. Gender and Health Research Unit, South African Medical Research Council, Pretoria, South Africa

3. School of Public Health, University of the Witwatersrand, Johannesburg, South Africa

**Corresponding author**:

Email address:

School of Nursing and Public Health, University of KwaZulu-Natal

Durban, South Africa

The discipline of Public Health Medicine, School of Nursing and Public Health, University of KwaZulu-Natal, Durban 4041, South Africa

# **Abstract**

Tuberculosis (TB) remains a leading cause of infectious disease mortality globally, with men in sub-Saharan Africa (SSA) disproportionately affected. Men experience higher TB incidence, delayed care-seeking, poorer treatment adherence, and elevated mortality compared to women. Gender-transformative health promotion interventions that challenge harmful masculine norms, enhance health system responsiveness, and foster positive health behaviours show promise for improving linkage to and retention in TB care. However, evidence on their design, implementation, and effectiveness in SSA is limited. Following the Arksey and O’Malley framework, refined by Levac et al., this scoping review will map evidence on gender-transformative health promotion interventions targeting adult men (≥18 years) diagnosed with any form of TB in SSA. We will search PubMed via MEDLINE, the Cochrane Library, Embase.com, Global Health, Web of Science, PsycINFO, Google Scholar, Africa-Wide Information, and the WHO Library, and screen reference lists of included studies. Eligibility will follow the Population–Concept–Context (PCC) framework, focusing on interventions supporting linkage to care, treatment initiation, adherence, or retention in care. Data will be charted using a standardized extraction tool, quality appraised using the Mixed Methods Appraisal Tool, Jadad scale, Newcastle–Ottawa Scale, and Joanna Briggs Institute checklists, and narratively synthesized using the RE-AIM (Reach, Effectiveness, Adoption, Implementation, Maintenance) framework. Stakeholder consultations will inform the interpretation of findings. As a protocol, no results are reported. The review will identify intervention characteristics, implementation strategies, barriers, facilitators, and outcomes across SSA contexts. Findings will guide the development of culturally sensitive, gender-responsive TB interventions aligned with the WHO End TB Strategy 2035 and Sustainable Development Goal 3 to improve linkage and retention among men diagnosed with TB in SSA.

**Registration:** *Open Science Framework.*

**License:** *CC0 1.0 Universal*

**Keywords:** *Tuberculosis, men’s health, masculinity, gender-transformative interventions, health promotion interventions, behavioral interventions, scoping review protocol, health equity, End TB Strategy 2035*.

**Background**

Tuberculosis (TB) remains one of the world’s deadliest infectious diseases, causing an estimated 1.25 million deaths annually and ranking just below Human Immunodeficiency Virus/Acquired Immunodeficiency syndrome (HIV/AIDS) in global mortality¹,². Despite advances in diagnosis and treatment, TB disproportionately affects low- and middle-income countries, particularly in sub-Saharan Africa (SSA)³,⁴.

Globally, TB incidence and mortality are consistently higher among men than women. In 2021, six million men developed TB compared to 3.4 million women, with men accounting for 54% of TB deaths among HIV-negative individuals in the African Region². In South Africa, pulmonary TB prevalence among men aged 15 years and older was 1.6 times higher than in women³,⁹, while a multi-country study across 56 nations reported a male-to-female TB incidence ratio of 1.8⁶.

Evidence from SSA further indicates that men are less likely than women to complete TB treatment and more likely to experience treatment default or failure³¹. For example, men had a 1.3–1.8 times higher risk of treatment interruption in multi-country SSA studies⁶,³², and in South Africa, men accounted for 60% of TB treatment interruptions with lower overall treatment completion rates compared to women⁹,¹⁴. Consequently, men face higher risks of TB complications and mortality despite the effectiveness of early diagnosis and treatment strategies²,¹⁴,⁴¹.

Gender disparities in TB outcomes arise from an interplay of biological, psychosocial, behavioral, and health system factors. Biological sex differences, including genetic and hormonal influences, affect immune responses and TB susceptibility¹⁶. Psychosocial determinants such as dominant masculine norms, imprisonment, homelessness, alcohol and substance use, smoking, and HIV co-infection, further elevate men’s risk¹.

Dominant masculine norms where entrenched ideals of stoicism and self-reliance hinder timely diagnosis, treatment initiation, and adherence⁸,³⁰ may discourage men from seeking care or expressing vulnerability, contributing to delayed diagnosis, poor treatment initiation, and high loss-to-follow-up rates²³,³². System-level barriers, including feminized healthcare environments, unwelcoming staff attitudes, and clinic hours incompatible with work schedules¹¹,⁸,⁵, also impede men’s engagement with TB services.

Nevertheless, global frameworks, including the World Health Organization (WHO) End TB Strategy⁴⁶, emphasize the need to implement gender-specific TB programmes²,⁴⁶. When combined with health promotion interventions that reduce stigma, increase access, and sustain engagement in care, these strategies enhance men’s participation across the TB care continuum¹,². In addition, co-developing interventions with men further improves cultural relevance, acceptability, and sustainability¹⁴,².

While progress in TB diagnostics and surveillance has advanced, equivalent investments in gender-sensitive programming remain insufficient, particularly in contexts where hegemonic masculinity norms impede care-seeking⁴⁴,¹. For instance, in SSA, TB interventions often remain gender-blind or gender-neutral, overlooking how masculinity shapes care-seeking behaviours²⁶,³⁹.

These disparities underscore the need for interventions that improve linkage, adherence, and retention specifically among men³. Indeed, emerging evidence suggests that gender-transformative interventions designed to challenge harmful masculine norms, foster supportive health systems, and promote positive health behaviours offer promise in improving TB outcomes among men²⁴,⁷,¹⁴. However, most evidence comes from high-income settings, and there is limited synthesized knowledge on the design, implementation, and outcomes of preventive and curative gender-transformative health promotion interventions for men with TB in SSA, including interventions that are sensitive to contextual and cultural factors and engage key actors involved in care delivery³⁸.

Given these challenges, mapping the design, implementation, and outcomes of gender-transformative health promotion interventions in SSA is essential to inform culturally and contextually appropriate strategies that enhance care engagement and treatment success among men³⁸. Along this line, a scoping review is warranted to map and synthesize existing evidence on the design, implementation, and outcomes of gender-transformative health promotion interventions aimed at improving linkage to and retention in care among adult men diagnosed with all forms of TB in SSA. This protocol article reports on the methods that will be used when conducting that scoping review.

**METHODS**

This scoping review forms part of a PhD study titled 'Developing a Gender-Transformative Health Promotion Intervention for Men Diagnosed with TB in the Eastern Cape Province, South Africa,' which has been approved by the Biomedical Research Ethics Committee (BREC) of the University of KwaZulu-Natal (BREC Ref No: 00008572/2025).

Reporting of the scoping review will adhere to the Preferred Reporting Items for Systematic Reviews and Meta-Analyses extension for Scoping Reviews (PRISMA-ScR)⁴⁰ to ensure methodological transparency and completeness (S1 Fig)

The scoping review’s methods are recorded according to the framework for scoping reviews proposed by Arksey and O’Malley² and refined by Levac et al.²² It includes six steps: 1) identifying the research question, 2) identifying relevant studies, 3) study selection, 4) charting the data, 5) collating, summarizing, and reporting results, and 6) consultation with stakeholders on study findings.

## **Identifying the research question**

A preliminary literature review was conducted to inform the development of a focused research question for the proposed scoping review. The central research question guiding this review is: What evidence exists on gender-transformative health promotion interventions that aim to strengthen linkage to care and retention in care among adult men diagnosed with TB in sub-SSA?

To address this overarching question, the review seeks to explore the following sub-questions:

1. What are the barriers and facilitators, including cultural and contextual factors, influencing gender-transformative health promotion interventions?
2. What methods, strategies, and delivery platforms are used to implement these interventions?
3. What are the outcomes of these interventions on linkage, treatment initiation, adherence, and retention in care?

## **Identifying relevant studies**

Relevant literature on men diagnosed with TB will be searched through electronic databases, including PubMed via MEDLINE, Cochrane Library, Embase.com, Global Health, Web of Science, PsycINFO, Google Scholar, Africa-Wide Information, and the WHO library database¹²,³⁴.

These databases were strategically selected for their relevance to the scope of this review, which focuses on gender-transformative health promotion interventions for men diagnosed with TB in sub-Saharan Africa¹². MEDLINE, Embase, Global Health, and the Cochrane Library were included due to their comprehensive coverage of biomedical and public health research, ensuring that clinical, epidemiological, and intervention studies relevant to TB management and health promotion were captured.

WHO Regional Libraries and Africa Wide Information were specifically selected to enhance geographic representation and provide access to region-specific studies and grey literature from high TB burden countries in sub-Saharan Africa. PsycINFO was included for its extensive coverage of psychological, behavioral, and mental health research, which is essential for understanding gendered behaviors, adherence patterns, and psychosocial components of health promotion interventions¹.

In addition to peer-reviewed publications, grey literature including government reports, policy briefs, and program evaluations will be included. These sources often provide valuable information on real-world implementation, contextual factors, and outcomes of TB interventions that may not be captured in peer-reviewed literature. Inclusion of grey literature ensures a more comprehensive mapping of gender-transformative interventions³⁸.

To ensure comprehensive coverage, the literature search will include studies published from 2000 to 2024. This extended timeframe allows the review to capture earlier interventions that may inform contemporary gender-transformative TB programs and identify changes in intervention design, implementation, and outcomes over time.

The preliminary search strategy (S2 File) was developed in consultation with a trained medical librarian (LR) to ensure thorough literature coverage. It will focus on combinations of keywords and controlled vocabulary related to key concepts such as “tuberculosis,” “men” or “male populations,” “gender-transformative interventions,” “health promotion,” and “sub-Saharan Africa”¹². This iterative strategy aims to capture a wide range of relevant studies, including biomedical, behavioral, and psychosocial research, aligning with the goals of this scoping review.

The principal investigator (SK), together with the primary supervisor, (YS), and the co-supervisor, (NK) will ensure the validity of the search strategy to be used.

**Study selection**

Study eligibility will be assessed by three reviewers who will first examine the titles and abstracts of relevant studies, and then their full texts. The reviewers will independently assess all potential studies using predefined inclusion criteria. To guide this process, we will apply the Population, Concept, and Context (PCC) framework recommended by Pollock et al.³⁵ and aligned with JBI guidelines¹⁹. The use of that framework will ensure that the eligibility criteria capture studies involving the relevant populations (adult men diagnosed with TB), focus on gender-transformative health promotion interventions, and are situated within appropriate contexts to answer the research questions (S3 Table 2).

For the context element, we will include studies conducted in countries within SSA that are classified as low- and middle-income countries (LMICs) according to the World Bank income classification at the time of the review. This approach ensures consistency with global health literature³ and transparency regarding the countries included. A complete list (S3 Table 1) of eligible countries is provided in the supplementary material.

## **Charting the data**

The next stage of the review involves data charting²²,³⁴,²⁷. Data from all included studies will be systematically extracted using a standardized charting tool developed by the review team (S4 File). Key information to be extracted includes: study type, location, participants’ sociodemographic characteristics (age, male gender, health status, and socio-economic status including level of self-rated health and health seeking behaviours among others), study purpose, design, setting where tuberculosis was diagnosed and treated (community versus hospital, Specialized TB centre or not), care providers in charge of tuberculosis at that centre (Specialist versus generalist…), presence or absence of anti-discrimination policies and tuberculosis gender-specific treatment policies in that setting, statistics from relevant inspection services on effective implementation of anti-discrimination policies and gender-specific tuberculosis treatment policies in that setting, type of tuberculosis assessed, tuberculosis treatment scheme and duration, implementation or not of complementary and alternative medicines in TB treatment scheme at that setting, direct and indirect costs of tuberculosis care at that setting, funder of tuberculosis treatment at that setting (government subsidies versus other payers including out-of-pocket payment by patients themselves), level of adherence to tuberculosis treatment, reasons for stopping tuberculosis treatment (e.g., death, loss to follow up…), barriers and facilitators to the implementation of health promotion interventions linking and/or retaining participants to care, health promotion intervention implemented, intervention type (preventive versus curative, gender-specific or not, culturally sensitive or not, informed by other local contextual issues or not), duration of follow up of participants, reasons for stopping the follow up, participants’ outcomes (survival versus hard outcomes such as quality of life, job opportunity, role conservation or modification in participants’ communities…).

A pilot test will be conducted on a sample of studies to refine the extraction categories and ensure clarity and consistency. Any discrepancies identified during the pilot will be resolved through discussion, and adjustments to the inclusion or extraction criteria will be made as necessary.

The principal investigator (SK) will lead the data extraction process, with the primary supervisor (YS) and co-supervisor (NK) independently verifying the extracted data for accuracy. The three authors will meet regularly throughout the extraction phase to review progress, resolve uncertainties, and ensure that the charting tool effectively captures all data relevant to the overarching research question.

The risk of bias of individual studies will be evaluated using the Mixed Methods Appraisal Tool (MMAT; S6 Fig) published in 2018¹³ to ensure data integrity, methodological rigor³⁶,²⁹,⁴² and quality, alignment with study aims, and appropriate implications for future research.

## **Collating, summarising, and reporting results**

We will employ a narrative synthesis approach to summarize descriptive data on gender-transformative health promotion interventions designed to strengthen linkages and retention of men diagnosed with TB in sub-Saharan Africa (SSA) .³ Study characteristics will be presented using tables and figures, stratified by key variables such as age, TB status, geographic location, and study design.

Although study quality will not be part of the eligibility criteria, we will appraise methodological quality using the Jadad scale for randomized trials¹⁸, the Newcastle-Ottawa scale for observational studies⁴³, and the Joanna Briggs Institute Critical Appraisal Tool for qualitative research¹⁹.

We will organize findings using Glasgow’s RE-AIM framework¹⁵ (Reach, Effectiveness, Adoption, Implementation, Maintenance). This will allow us to assess study reach, methodological diversity, diagnostic and intervention approaches, adoption into practice and policy, implementation of barriers and facilitators, and sustainability within health systems. Knowledge Gaps in the literature will be identified through comparative analysis to inform future research priorities and the development of a gender-transformative intervention framework³⁸.

## **Consultation with stakeholders on study findings**

The consultation phase will engage healthcare workers, TB experts, stakeholders, and men with lived experience of TB to ensure findings are contextually relevant and actionable³⁸ and can guide future locally tailored, effective strategies. This review will examine culturally sensitive features of interventions and the roles of key actors including healthcare providers, policymakers,and men with lived experience in their design and implementation, highlighting insights that.

**Definition of Terms**

To ensure methodological rigor and consistency, the following key terms are defined based on recent evidence²¹,²:

- **Tuberculosis (TB):** A bacterial infectious disease caused by Mycobacterium tuberculosis, primarily affecting the lungs but capable of affecting other organs. For this scoping review, TB includes all forms diagnosed among adult men, specifically: pulmonary TB (microbiologically confirmed or clinically diagnosed), extra-pulmonary TB, latent TB, active TB, drug-sensitive TB, and drug-resistant TB.
- **Gender:** Socially constructed characteristics, norms, roles, and behaviors associated with women, men, girls, and boys. Gender is context-specific, dynamic, and influences health behaviors and access to care.
- **Health:** A state of complete physical, mental, and social well-being, not merely the absence of disease or infirmity.
- **Health Promotion:** The process of enabling individuals and communities to increase control over determinants of health and improve overall well-being.
- **Linkage to Care:** The initiation of engagement with TB services following diagnosis, typically operationalized as attending a first clinical visit within 5–15 days post-diagnosis.
- **Retention in Care:** Ongoing, regular engagement with TB clinical services after initial linkage, encompassing adherence to treatment schedules and follow-up appointments.
- **Gender-Transformative Interventions:** Strategies that explicitly address and aim to change harmful gender norms and behaviors, improve health system responsiveness, and promote equitable access and sustained engagement in care.

## **Data Sharing**

In line with open science principles, the final data extraction sheet and metadata will be made publicly available via the Open Science Framework³⁸.

## **Timeline**

The initial search will be conducted on October 31, 2025. Title and abstract screening, along with full-text screening, will be carried out from November 2025 to January 2026. Data extraction will be completed by March 2026, followed by analysis and synthesis of results from March to May 2026. As of September 2026, we anticipate finalizing the results and submitting the manuscript for publication by June 2026.

## **Ethical Considerations**

Ethical approval was not required for this scoping review, as it involves the synthesis of data from published literature and does not include human participants². The study will be conducted in accordance with the principles of the Declaration of Helsinki and the ethical guidelines of the Biomedical Research Ethics Committee (BREC) of the University of KwaZulu-Natal (UKZN), ensuring integrity, transparency, and responsible handling of research evidence.

# **DISCUSSION**

This scoping review will map and synthesize existing literature on gender-transformative and health promotion interventions in sub-Saharan Africa (SSA) designed to improve linkage to care and retention among men aged 18 years and older diagnosed with TB³,⁴⁶. The review aligns with the WHO’s post-2015 End TB Strategy, adopted by the World Health Assembly in 2014 and embedded within the Sustainable Development Goals²⁰,⁴⁶.

The proposed methods offer several anticipated strengths. Their structured and iterative approach is expected to provide a comprehensive overview of current interventions while maintaining transparency in study selection and data extraction²,²². Drawing on diverse data sources, engaging stakeholders, and employing robust quality appraisal tools will enhance the depth and contextual relevance of the findings¹³,¹⁵. Moreover, the use of a well-established synthesis framework, such as RE-AIM¹⁵, will support the generation of practical insights to inform future intervention design and policy development.

Nonetheless, certain methodological limitations are expected. Restricting the search to published and English-language studies may introduce selection bias and omit relevant evidence³⁸. The heterogeneity of intervention designs and outcomes is also likely to limit comparability and preclude quantitative synthesis. As with most scoping reviews, the focus will be on mapping rather than critically evaluating intervention effectiveness, which may constrain the strength of resulting policy recommendations.

To address these limitations, mitigation strategies such as iterative team discussions, piloting of data extraction tools, and triangulation with stakeholder perspectives will be employed, thus improve the reliability and applicability of the review findings³⁴,³⁸.

**Dissemination Plan**

The findings of this scoping review will be submitted to PLOS ONE for publication. Additionally, the results will be shared through institutional websites and professional networks such as LinkedIn. Where feasible, preliminary findings will be presented at relevant conferences and meetings to engage the broader research and public health community³⁸.

**Conclusion**

This scoping review will advance understanding of how gender-transformative and health promotion interventions have been designed, implemented, and evaluated to improve linkage to care and retention among men diagnosed with tuberculosis in sub-Saharan Africa³. By systematically mapping existing evidence, assessing intervention outcomes, and highlighting key knowledge gaps, the review will generate insights to guide the development of context-specific, gender-responsive strategies and inform future research, policy, and programmatic efforts aligned with the WHO End TB Strategy²,⁴⁶ and Sustainable Development Goals.

**Supporting Information**

**S1 Fig** PRISMA FLOW checklist

**S 2 Fig** initial search terms by database

**S3 Table 1** Table Sub-Saharan African Countries Classified as LMICs (2025)

**S4 Table 2** Population, Concept, and Context

**S 5 Fig** DATA CHARTING form

**S 6** **Fig** MIXED METHOD APPRAISAL tool

# **Declarations**

## **Consent for Publication**- Not Applicable

## **Availability of the data materials-** Not applicable

## **Competing Interests-** The authors declare that they have no competing interests.

## **Funding**

This research received no specific grant from any funding agency in the public, commercial, or not-for-profit sectors.

## **Authors Contribution**

SK, YS and NK led the conceptualization and design of this scoping review. SK,

YS and NK each made substantial contributions to the drafting and critical

revision of the work and all authors approved the final manuscript.

## **Acknowledgements**

Our gratitude goes to the School of Nursing and Public Health, Discipline of Public Health, University of KwaZulu-Natal for guidance and support in developing this scoping review protocol.

# **References**

1. Allwood BW, Nightingale R, Agbota G, Auld S, Bisson GP, Byrne A, et al. Perspectives from the 2nd International Post-Tuberculosis Symposium: Mobilising advocacy and research for improved outcomes. IJTLD Open. 2024;1(3):111–23.
2. Arksey H, O’Malley L. Scoping studies: Towards a methodological framework. Int J Soc Res Methodol. 2005;8(1):19–32.
3. Brown C, Patel V, Mabaso M, Dlamini N. Improving treatment adherence among men with tuberculosis: Lessons from behavioural interventions in low-resource settings. J Health Psychol. 2019;24(12):1801–12.
4. Centers for Disease Control and Prevention (CDC). Global tuberculosis report 2025. Atlanta: CDC; 2025.
5. Chikovore J, Hart G, Kumwenda M, Chipungu GA, Desmond N. “TB is not a woman’s disease”: Exploring gendered stigma and care-seeking behaviours. Soc Sci Med. 2024;345:115383.
6. Chikovore J, Hart G, Kumwenda M, Chipungu GA, Desmond N. TB and masculinity in sub-Saharan Africa: Implications for health promotion. Glob Health Action. 2020;13(1):1797573.
7. Colvin CJ. Gender, health and TB: Understanding transformative programming. BMC Public Health. 2019;19(1):1235.
8. Daniels J, Kliner M, Naidoo P, Naidoo K. Gendered dimensions of TB care in KwaZulu-Natal: Understanding access barriers and facilitators. BMC Health Serv Res. 2021;21(1):1–10.
9. Dhlakama A, Naidoo K, Zuma N, Naidoo P. Gender differentials in tuberculosis treatment outcomes in South Africa: A cohort analysis. Int J Tuberc Lung Dis. 2022;26(11):1052–9.
10. Diwan VK, Thorson A. Sex, gender, and tuberculosis. Lancet. 1999;353(9157):1000–1.
11. Fleming PJ, Lee JG, Dworkin SL. “Real men don't”: Constructions of masculinity and inadvertent harm in public health interventions. Am J Public Health. 2016;106(7):1188–96.
12. Foo CD, Shrestha P, Wang L, Du Q, García-Basteiro AL, Abdullah AS, et al. Integrating tuberculosis and noncommunicable diseases care in low- and middle-income countries (LMICs): a systematic review. PLoS Med. 2022;19(1):e1003899.
13. Gavallo C, Hong QN, Pluye P. Applying the Mixed Methods Appraisal Tool (MMAT) 2018 in health research: Practical guidance. J Clin Epidemiol. 2023;158:1–10.
14. Gibbs A, Vaughan C, Aggleton P. Transforming masculinities: Using gender-transformative approaches to improve health and wellbeing. BMC Public Health. 2019;19(1):1463.
15. Glasgow RE. RE-AIM framework: Translating research into practice. Am J Public Health. 1999;89(9):1322–7.
16. Gupta S, Kakkar R, Sharma R, Gupta M. Sex-based differences in immune responses and tuberculosis susceptibility: Implications for diagnosis and treatment. Front Immunol. 2022;13:987654.
17. Horton KC, MacPherson P, Houben RM, White RG, Corbett EL. Sex differences in tuberculosis burden and notifications in low- and middle-income countries: a systematic review and meta-analysis. PLoS Med. 2016;13(9):e1002119.
18. Jadad AR, Moore RA, Carroll D, Jenkinson C, Reynolds DJ, Gavaghan DJ, et al. Assessing the quality of reports of randomized clinical trials: Is blinding necessary? Control Clin Trials. 1986;17(1):1–12.
19. Joanna Briggs Institute (JBI). Critical appraisal checklist for qualitative research. Adelaide: JBI; 2019.
20. Kuupiel D, Tlou B, Mashamba-Thompson TP. The role of health systems in achieving the WHO End TB Strategy targets: A scoping review. BMC Health Serv Res. 2019;19(1):189.
21. Ledesma JR, Ma J, Zhang M, Basting AV, Chu HT, Vongpradith A, et al. Global, regional, and national age-specific progress towards the 2020 milestones of the WHO End TB Strategy: a systematic analysis for the Global Burden of Disease Study 2021. Lancet Infect Dis. 2024;24(7):698–725.
22. Levac D, Colquhoun H, O’Brien KK. Scoping studies: Advancing the methodology. Implement Sci. 2010;5(1):69.
23. MacPherson P, Houben RM, Glynn JR, Corbett EL, Kranzer K. Pre-treatment loss to follow-up in tuberculosis patients: A systematic review and meta-analysis. Int J Tuberc Lung Dis. 2015;18(12):1509–18.
24. Majid F, Colvin CJ, Gibbs A. Gender-transformative interventions and men’s engagement in health: A review of evidence. Cult Health Sex. 2018;20(10):1119–36.
25. Marçôa R, Ribeiro AI, Zão I, Duarte R. Tuberculosis and gender – Factors influencing the risk of tuberculosis among men and women by age group. Pulmonology. 2018;24(3):199–202.
26. Medina-Marino A, Huerga H, Boyles T. Understanding masculinity in TB care engagement in Africa: A policy perspective. Afr J Prim Health Care Fam Med. 2025;17(1):e158–67.
27. Monteiro L, Henley J, Silva L, Dlamini S. Charting data in scoping reviews: Lessons from health promotion research. BMC Med Res Methodol. 2024;24(1):88.
28. Montgomery AL, Khan FA, Denholm JT, Fox GJ. The global epidemiology of tuberculosis: Past, present, and future. Clin Chest Med. 2016;37(4):425–45.
29. Morrison C, Sweeney K, Jones E. Mixed-methods research in health: Evaluating quality and outcomes. Health Serv Res. 2011;46(6):1825–39.
30. Mosiwa B, Chimbindi N, Nzimande K. Masculinities and health in Africa: How gender norms influence TB treatment adherence. Health Promot Int. 2025;40(2):723–34.
31. Mphothulo T, Mashabela G, Khoza S. Gender disparities in tuberculosis treatment outcomes in Southern Africa: A multi-country cohort study. BMC Public Health. 2025;25(1):188.
32. Naidoo P, Theron G, Rangaka MX, Chihota VN, Vaughan L, Brey Z, et al. The South African tuberculosis care cascade: Estimated losses and methodological challenges. J Infect Dis. 2017;216(suppl_7):S702–13.
33. National Department of Health (NDoH). National tuberculosis prevalence survey, South Africa, 2017–2019. Pretoria: NDoH; 2021.
34. Peters MDJ, Godfrey CM, McInerney P, Munn Z, Tricco AC, Khalil H. Chapter 11: Scoping reviews (2020 version). In: JBI Manual for Evidence Synthesis. Adelaide: Joanna Briggs Institute; 2020.
35. Pollock D, Davies EL, Peters MDJ, Tricco AC, Alexander L, McInerney P, et al. Developing scoping review methods: An update on JBI guidance. JBI Evid Synth. 2023;21(2):202–12.
36. Prasanna K, Thapa P, Moyo S. Appraising mixed-methods studies in public health: A methodological review. Glob Health Res Policy. 2024;9(1):24.
37. Rine R, Modisenyane T, Ndlovu N, Mthembu S. Understanding TB program outcomes in sub-Saharan Africa: A review of implementation challenges. Afr Health Sci. 2023;23(1):33–46.
38. Sharp N, Mash R, Naidoo P. Health promotion and TB care in African contexts: Lessons from participatory approaches. Health Educ J. 2025;84(2):189–206.
39. Tricco AC, Lillie E, Zarin W, O’Brien KK, Colquhoun H, Levac D, et al. PRISMA extension for scoping reviews (PRISMA-ScR): Checklist and explanation. Ann Intern Med. 2018;169(7):467–73.
40. UNAIDS. Tuberculosis and gender: Breaking the barriers to care. Geneva: UNAIDS; 2018.
41. Waghule PR, Patil K, Marathe S. Quality assessment tools for systematic reviews: A comparative analysis. J Evid Based Med. 2021;14(2):87–96.
42. Wells GA, Shea B, O'Connell D, Peterson J, Welch V, Losos M, et al. The Newcastle–Ottawa Scale (NOS) for assessing the quality of nonrandomised studies in meta-analyses. Ottawa Hospital Research Institute; 2021.
43. World Health Organization (WHO). Global tuberculosis report 2023. Geneva: WHO; 2023.
44. World Health Organization (WHO). Global tuberculosis report 2024. Geneva: World Health Organization; 2024.
45. World Health Organization (WHO). The End TB Strategy. Geneva: WHO; 2015.
