# Supplementary material for: Gender-transformative health promotion interventions for linking and retaining tuberculosis-diagnosed adult men in care in sub-Saharan Africa: A scoping review protocol
Source: PLoS One. 2026 Jan 8;21(1):e0339666. doi: 10.1371/journal.pone.0339666 (PMC12782366; doi:10.1371/journal.pone.0339666)
Supplement: Attachment — Submitted filename: Response to reviewers2.docx [file pone.0339666.s011.docx]

# **Query response sheet**

**Decision letter specific comments from the editor**

| **No.** | **Query** | **Response to Query** | **Original Wording (if applicable)** |
| --- | --- | --- | --- |
| 1 | Gender-transformative health promotion interventions for linking and retaining tuberculosis-diagnosed adult men (18 years and above) in care in sub-Saharan Africa: A scoping review protocol. | Thanks to the reviewer to the advice, we have accepted the removal of the bracketed words (18 years and above) in the corrected version of the manuscript. The new topic is as follows:  Gender-transformative health promotion interventions for linking and retaining tuberculosis-diagnosed adult men in care in sub-Saharan Africa: A scoping review protocol. |  |
| 2 | Findings will guide the development of culturally sensitive, gender-responsive TB interventions aligned with the WHO End TB Strategy 2025 and Sustainable Development Goal 3 to improve linkage and retention among men diagnosed with TB in SSA. | Thanks to the reviewer for highlighting this error; we have accepted the insertion of 2025 in the WHO End TB Strategy to bring clarity to the exact policy. |  |
| 3 | As mentioned in the first round of peer review, consider providing the good reference number of your protocol on OSF here. The reference number you had provided us with in the first round of review rather corresponds to a scoping review protocol titled «Mapping factors influencing initiation of antiretroviral treatment among adolescents living with HIV/AIDS in sub-Saharan Africa: A scoping review protocol, which is different from the title of this manuscript and from the title of your thesis mentioned in the Methods section. | Thanks to the reviewer for bringing this up, we have removed sentence and replaced it with an appropriate DOI generated from the OSF system as follows:  10.17605/OSF.IO/7EHR9 | Registration: Open Science Framework. |
| 4 | This one is not warranted.”  License: CC0 1.0 Universal | We appreciate the suggestion. The sentence has now been removed |  |
| 5 | Does the reference by Arksey and O’Malley support this statement? | Thank you for highlighting this. Upon review, we recognized that the statement was not directly supported by Arksey and O’Malley. We have therefore removed this reference and revised the sentence accordingly to ensure the claim is supported by appropriate and more relevant sources. |  |
| 6 | Despite advances in diagnosis and treatment, TB disproportionately affects low- and middle-income countries, particularly in sub-Saharan Africa (SSA)³,⁴. | Thank you for this helpful suggestion. We have now added the abbreviation “SSA” after the first mention of Sub-Saharan Africa and used it consistently throughout the manuscript to improve clarity and readability. |  |
| 7 | Consider moving the tables on preliminary search strategies to the supplementary material…” | Thank you for this helpful suggestion. In line with guidance for transparent reporting in scoping reviews (PRISMA-ScR and JBI), the full PubMed search strategy table has been added to the Methods section (Page 7, Table 1). All other database search strategies are retained in S2 File. |  |
| 8 | “There is nowhere in this subsection where you refer readers to Table 1…” | Thank you for this comment. We have revised the text to explicitly guide readers to the table where the detailed inclusion and exclusion criteria are presented. Specifically, we now refer readers to S4 Table 3, which contains the full eligibility criteria organized according to the Population, Concept, and Context (PCC) framework.  Thhe revised passage reads as follows:  “To guide the study selection process, we will apply the Population, Concept, and Context (PCC) framework as recommended by Pollock et al.³⁵ and in alignment with JBI guidelines¹⁹. This framework ensures that the eligibility criteria capture studies involving the relevant population (adult men diagnosed with TB), focus on gender-transformative health promotion interventions, and are situated within appropriate contexts to address our research questions. Detailed inclusion and exclusion criteria can be found in S4 Table 3.”  This change ensures readers can directly locate the criteria used for study selection. |  |
| 9 | Why three individuals? Can you put the initials of their names in brackets if they are authors of this manuscript? Will they perform independent study selection? How are they going to handle disagreements about study selection? All these precisions are needed. | Thank you for this thoughtful comment. We have revised the manuscript to clarify the roles of the reviewers involved in study selection. As now indicated, two reviewers (SK and YS) will independently conduct both the title/abstract screening and full-text screening using the predefined eligibility criteria. A third reviewer (NK) is included to ensure methodological rigor and will act as an adjudicator in cases where consensus cannot be reached between the primary reviewers. This approach aligns with recommended scoping review methodology, which emphasizes independent screening by at least two reviewers to minimize bias and enhance reliability. The revised text now specifies the reviewers by initials, confirms that study selection will be conducted independently, and outlines the process for resolving disagreements. |  |
| 10 | “You need to provide a reference supporting this statement.” | Thank you for your comment. We have added a reference to support the statement regarding the inclusion of studies conducted in Sub-Saharan African countries classified as low- and middle-income by the World Bank. Specifically, we now cite the World Bank’s country and lending group classifications, which provide the authoritative source for identifying low- and middle-income countries.  For example, the revised sentence reads:  “Studies conducted in countries within Sub-Saharan Africa that are classified as low- and middle-income according to the World Bank income classification will be included ^46^. |  |
| 11 | Normally, this statement should be moved to the PCC framework table.” | Thank you for the suggestion. We appreciate the reviewer’s attention to the appropriate placement of this statement. The statement describing the context element—specifically the inclusion of studies conducted in sub-Saharan African countries classified as LMICs according to the World Bank classification—has been moved to the PCC (Population–Concept–Context) framework table as recommended. This adjustment ensures clarity, alignment with the framework’s structure, and consistency in the presentation of inclusion criteria. |  |
| 12 | “Is this sentence necessary? I would delete it.” | Thank you for your thoughtful observation. We agree that the sentence is not essential for the clarity of the manuscript. We have therefore removed it as suggested. We appreciate your guidance in helping to improve the conciseness and readability of the text. | The next stage of the review involves data charting |
| 13 | Gender or sex? Cis gender men or transgender men? Are there not transgender and non-binary people in sub-Saharan Africa? See https://doi.org/10.1186/s44263-024-00073-2 | Thank you for this important clarification. In our review, we use the term “gender” rather than “sex” because our focus is on the socially constructed roles, behaviours, and norms that influence health-seeking practices and engagement in TB care. The review specifically targets cisgender men, as most of the included studies and available data in sub-Saharan Africa refer to this population. We have revised the manuscript to clarify this distinction and explicitly note that transgender men are not the primary focus due to the limited availability of disaggregated data in the included studies. |  |
| 14 | “Consider implementing all these variables in the data extraction sheet.” | Thank you very much for your valuable and constructive feedback on the Data Charting Tool. We sincerely appreciate your careful review and guidance.  In response, we have revised the data charting tool to incorporate all the suggested variables, the updated version has been attached as a supporting document. We think that the revision of the tool will make the scoping review to be comprehensive, detailed, and aligned with objectives. |  |
| 15 | This statement belongs to the preceding sub-section on study selection. | Thank you for noting that. We have removed the statement from this section and relocated it to the preceding sub-section on Study Selection for better logical flow and consistency. | Any discrepancies identified during the pilot will be resolved through discussion, and adjustments to the inclusion or extraction criteria will be made as necessary. |
| 16 | Consider avoiding repetition in the manuscript, because this sentence reiterates the study’s aim which is already present in the introduction. You could just write: We will employ A narrative synthesis Approach. | Thank you for this helpful suggestion. We agree that the sentence was repetitive, as the study aim is already clearly stated in the introduction. We have revised the text accordingly and now simply state that we will employ a narrative synthesis approach. We appreciate your careful reading and guidance in improving the clarity of the manuscript. | We will employ a narrative synthesis approach to summarize descriptive data on gender-transformative health promotion interventions designed to strengthen linkages and retention of men diagnosed with TB in sub-Saharan Africa (SSA) |
| 17 | Did you not say above that you will use the MMAT tool?” | Thank you for drawing attention to this inconsistency. You are correct that we stated in the previous section that the methodological quality of all included studies would be appraised using the Mixed Methods Appraisal Tool (MMAT). The sentence referring to the use of the Jadad scale, Newcastle–Ottawa scale, and JBI tools reflects an earlier version of the protocol and was not updated when we moved to using MMAT as a single, unified appraisal tool. We have now removed that sentence to ensure consistency and clarity. We appreciate your careful reading and guidance in helping us maintain coherence in the manuscript. | Although study quality will not be part of the eligibility criteria, we will appraise methodological quality using the Jadad scale for randomized trials¹⁸, the Newcastle-Ottawa scale for observational studies⁴³, and the Joanna Briggs Institute Critical Appraisal Tool for qualitative research |
| 18 | Comparative analysis with what? Specify.” | We clarified that comparative analysis will occur across study settings, intervention types, and outcomes, to identify patterns and gaps. |  |
| 19 | I would remove this statement which is just a repetition of the previous sentence when looking at it carefully. | Thank you for pointing this out. Upon review, we agree that the sentence was redundant and did not add new information. We have removed it to improve clarity and avoid repetition. We appreciate the careful reading and helpful suggestion. |  |
| 20 | “Does this reference help define any of these terms?” | Thank you for this observation. We have revised the definitions accordingly and strengthened this section by incorporating verbatim definitions from authoritative sources, including UNESCO, the World Health Organization (WHO), and other reputable institutions. These updates ensure accuracy, consistency with global standards, and improved clarity in the conceptualisation of key terms. | To ensure methodological rigor and consistency, the following key terms are defined based on recent evidence²¹,²: |
| 21 | Should be in Italic.” | Thank you for noting this formatting issue. We have now amended the manuscript and ensured that the relevant text appears in italic as requested. | Mycobacterium tuberculosis |
| 22 | “This sentence is not warranted…” | Thank you for your observation. We agree that the sentence is unnecessary given that the information is already clearly presented in the eligibility criteria table. We have removed the sentence to avoid redundancy and improve the clarity of the manuscript. | . For this scoping review, TB includes all forms diagnosed among adult men, specifically: pulmonary TB (microbiologically confirmed or clinically diagnosed), extra-pulmonary TB, latent TB, active TB, drug-sensitive TB, and drug-resistant TB. |
| 23 | I guess reference 21only supports the definition of tuberculosis. So, mention it where you define tuberculosis only | Thank you for this helpful clarification. We agree with your observation. In addition, we have strengthened this definition by extracting it verbatim from the 2024 WHO report to ensure accuracy and alignment with authoritative guidance. |  |
| 24 | Look for WHO references to back up these definitions and make verbatim transcriptions of WHO statements for these definitions. | Thank you for the feedback. We have revised the definitions accordingly and strengthened this section by incorporating verbatim definitions from authoritative sources, including UNESCO, the World Health Organization (WHO), and other reputable institutions. These updates ensure greater accuracy, alignment with global standards, and improved clarity in the conceptualisation of key terms. |  |
| 25 | Given the plan to submit the scoping review for publication y June next year, consider modifying the expected date for the finalization of data analysis and interpretation | Thank you for this helpful suggestion. We have revised the timeline accordingly to ensure better alignment with the planned manuscript submission date in September 2026. These adjustments provide a more realistic and coherent timeline for completing the review and preparing a high-quality submission. |  |
| 26 | Just before this sub-section, insert another sub-section titled Protocol deviation. See how the others did that: https://doi.org/10.1371/journal.pone.0328645 | Thank you for your suggestion. We have inserted a new subsection titled “Protocol deviation” just before the Ethical consideration subsection. This mirrors the structure used in the referenced article (doi:10.1371/journal.pone.0328645) and enhances the clarity and transparency of our methods section. |  |
| 27 | “Is this citation gaming? Does reference 38 exist?” | Thank you for bringing this to our attention. Upon review, we realized that reference 38 does not exist. We have now removed the citation and ensured that the statement is supported by existing references |  |
| 28 | H ave you not s aid you would focus on qualitative narrative synthesis? When mentioning limitations, have you highlighted the potential lack of representation of some subcontinental regions, especially since this is not a topic assessed in everyday health research. | Thank you for your observation. We acknowledge the error in suggesting the potential for quantitative synthesis. As outlined in our protocol, this scoping review will focus on a qualitative narrative synthesis due to the anticipated heterogeneity of interventions and outcomes. We have revised the manuscript to clarify this point and to highlight that the review emphasizes mapping and synthesizing evidence rather than conducting quantitative comparisons. |  |
| 29 | Is this the correct reference for number 2? | Thank you for pointing this out. Upon review, we acknowledge that reference number 2 was incorrectly cited due to a typographical error. We have corrected the reference to ensure accuracy in the manuscript. |  |
| 30 | I Health Educ J. 2025;84(2):189–206.m un Health Educ J. 2025;84(2):189–206.ble to find this reference online, even on Google. | Thank you for your vigilance. We have attempted to trace the cited article, but we found no evidence that it exists in any major database or repository. Therefore, we will remove the reference from the manuscript to maintain the integrity of our citations. | Sharp N, Mash R, Naidoo P. Health promotion and TB care in African contexts: Lessons from participatory approaches. Health Educ J. 2025;84(2):189–206. |

**Plos one academic editor’s additional general comments**

| **No.** | **Query** | **Response to Query** | **Original Wording (if applicable)** |
| --- | --- | --- | --- |
| 1 | I have proposed a deletion of the statement (18 years and above) in the manuscript’s title to keep it shorter. | Thanks to the reviewer to the advice, we have accepted the removal of the bracketed words (18 years and above) in the corrected version of the manuscript. The new topic is as follows:  Gender-transformative health promotion interventions for linking and retaining tuberculosis-diagnosed adult men in care in sub-Saharan Africa: A scoping review protocol. |  |
| 2 | I have have proposed a reorganization of the introduction to improve its flow. | Thank you for your thoughtful suggestion. We appreciate the proposed reorganization of the introduction, and we agree that it improves the logical flow and clarity of the manuscript. We have revised the introduction accordingly to incorporate these changes. |  |
| 3 | I also proposed edits for the Methods and Discussion sections. | Thank you for your valuable suggestions. We have incorporated the proposed edits to the Introduction, Methods, and Discussion sections, which have enhanced the clarity and flow of the manuscript. |  |
| 4 | To avoid redundancy in the manuscript, I have proposed a deletion of the statement «To ensure a comprehensive coverage of the published literature, the review will include studies published from 2000 to 2024, allowing the capture of earlier interventions that may inform contemporary programs and assess changes in intervention design, implementation, and outcomes over time. This review will also explore contextual, cultural, and structural factors influencing intervention uptake and the role of key actors involved in intervention design and delivery.» in the introduction, leaving it only in the Methods section. | Thank you for your suggestion. To avoid redundancy and improve the flow of the manuscript, we have deleted the statement from the Introduction and retained it in the Methods section, where it provides the most appropriate context for the study design and scope. |  |
| 5 | The text states that you included search terms related to sub-Saharan Africa in the search strategy. However, there are no terms related to sub-Saharan Africa in any table on search strategies provided in the supplemental material. Consider addressing that issue. | Thank you for pointing this out. We have addressed the issue by adding the missing key term related to sub-Saharan Africa in both the search strategy table in the supplemental material and the keywords listed in the manuscript. This ensures consistency between the text and the documented search strategy and improves the transparency and reproducibility of our review. |  |
| 6 | You seem to have implemented the eligibility criteria proposed in the first round of peer review. However, it is difficult to read your eligibility criteria, and there is inconsistency with the eligibility criteria currently listed, at least for the type of publications that will be excluded. As suggested in the first round of peer review, consider going through this article (https://doi.org/10.1371/journal.pone.0322753) and build your table on eligibility criteria exactly as they did to improve its readability. | Thank you very much for this helpful observation and for directing us to the example article. We appreciate the reviewer’s attention to clarity and consistency in the presentation of our eligibility criteria. In response, we have thoroughly revised and reformatted the eligibility criteria table following the structure and style used in the suggested PLOS ONE article. The updated table now presents inclusion and exclusion criteria side by side under clearly defined domains (Population, Concept, and Context), using concise and parallel wording to improve readability. We have also harmonized the types of excluded publications across all domains to ensure internal consistency. Additionally, the “Context” domain now includes a cross-reference to the annexure table listing all low- and middle-income countries (LMICs) classified by the World Bank during the study period. |  |
| 7 | Consider also rearranging tables’ numbers in the manuscript and in the supplementary material. | Thank you for this helpful suggestion. We have reviewed the tables in both the main manuscript and the supplementary material and have rearranged and renumbered them to ensure consistency, clarity, and alignment throughout the document. |  |
| 8 | Notably, consider inserting all tables (including those from the supplemental material) in a Word format rather than in a TIF format as seen in the current manuscript. | Thank you for bringing this to our attention. We have processed all tables, including those in the supplementary material, and converted them from TIF format to Word-compatible formats. This ensures improved accessibility, consistency, and ease of review in line with journal requirements. |  |
| 9 | Consider updating references in the text and formatting references in the text and the reference section as observed in published PLOS One articles. | Thank you for this helpful suggestion. We have carefully reviewed the PLOS ONE reference guidelines and examined formatting used in recently published PLOS ONE articles. In response:  All in-text citations have been updated to follow PLOS ONE’s numerical citation style, ensuring consistency throughout the manuscript.  The reference list has been reformatted according to PLOS ONE requirements, including adherence to Vancouver style, correct ordering, punctuation, journal abbreviations, and inclusion of DOIs where applicable.  Outdated or non-compliant references were updated or corrected to align with current PLOS ONE formatting standards.  We appreciate the reviewer’s guidance, which has improved the clarity and compliance of the manuscript. |  |
| 10 | Furthermore, there is some degree of citation gaming in this manuscript, and this issue should be addressed as well. | Thank you for raising this important concern. We take issues related to citation integrity very seriously. In response, we undertook a careful review of all citations to ensure that:  All referenced works directly support the statements made in the manuscript and are clearly relevant to the topic.  Non-essential, tangential, or potentially self-promotional citations were removed to avoid any perception of citation inflation.  The revised reference list includes only studies that contribute substantively to the background and method section of the protocol.  We appreciate the reviewer’s attention to this matter. The revised manuscript now reflects a more balanced and rigorous citation approach aligned with scholarly and ethical standards. |  |
| 11 | Along this line, you keep mentioning the WHO End TB strategy throughout the manuscript whereas the period of evaluation of that strategy (2015 to 2020 is clearly over (see Lancet Infect Dis 2024; 24: 698–725), and reference 46 they used to back up their claims on the WHO End TB strategy is missing at the end of the manuscript. Therefore, consider removing all comments on the WHO End TB strategy in this manuscript. | Thank you for this valuable observation. We agree that the WHO End TB Strategy’s primary evaluation period (2015–2020) has concluded, and the Lancet Infectious Diseases analysis you cited provides updated evidence and context. In response:  We have removed all references to the WHO End TB Strategy that implied ongoing evaluation or relevance to the current period.  Mentions of the strategy have been substantially reduced, and retained only where historically necessary (e.g., when describing legacy frameworks or contextual background), but without positioning it as an active guiding framework.  The missing reference previously cited as Reference 46 has been removed, and the associated claim has been revised to avoid unsupported statements.  The manuscript has been updated to reflect more current policy and programmatic frameworks where relevant, ensuring temporal accuracy and alignment with recent TB program literature.  We appreciate the reviewer highlighting this issue, which has strengthened the accuracy and currency of the manuscript. |  |
