# Supplementary material for: Gender-transformative health promotion interventions for linking and retaining tuberculosis-diagnosed adult men in care in sub-Saharan Africa: A scoping review protocol
Source: PLoS One. 2026 Jan 8;21(1):e0339666. doi: 10.1371/journal.pone.0339666 (PMC12782366; doi:10.1371/journal.pone.0339666)
Supplement: Attachment — Submitted filename: Response to reviewers3.docx [file pone.0339666.s012.docx]

# **Query response sheet**

**Decision letter specific comments from the editor**

| **No.** | **Query** | **Response to Query** | **Original Wording (if applicable)** |
| --- | --- | --- | --- |
| 1 | In the hindsight, I came to realise that the WHO end TB strategy is ongoing, although ref 46 in the last version of the manuscript was misleading. See <Https://www.who.int/teams/global-programme-on-tuberculosis-and-lung-health/the-end-tb-strategy>. | Thank you for highlighting this important point. We acknowledge that the WHO End TB Strategy is an ongoing initiative. In the earlier version of the manuscript, the way reference 46 was cited may have unintentionally created ambiguity. Given the substantial revisions already made throughout the manuscript and to avoid disrupting the coherence of the revised structure, we have opted not to reintegrate the End TB Strategy into the current version. We appreciate the editor’s attention to this detail and confirm that the manuscript has been adjusted to remove any misleading phrasing. |  |
| 2 | Careful English language editing is required, removing typos and other language mistakes. For instance, what is the abbreviation of aub-Saharan Africa in this manuscript SSA or sub-SSA? | Thank you for pointing this out. We have carefully reviewed the entire manuscript to correct typographical errors, improve clarity, and ensure consistent use of terminology. Specifically, we standardized the abbreviation for sub-Saharan Africa to “SSA” throughout the manuscript and removed any inconsistent forms such as “sub-SSA.” We appreciate the editor’s attention to these language issues, which has helped strengthen the overall quality and coherence of the manuscript. |  |
| 3 | Conform to PLOS ONE referencing system. | We thank the editor for this important note. We have carefully reviewed the manuscript and revised all references to conform to the PLOS ONE referencing style, ensuring consistency and accuracy throughout the text and reference list. |  |
| 4 | The search strategy should include the complete list of SSA countries based on the World Bank classification. See for example systematic reviews on other conditions in SSA. | Thank you very much for this helpful suggestion. We agree that including the full list of Sub-Saharan African (SSA) countries, based on the World Bank’s regional and income classification, will strengthen the transparency and reproducibility of our search strategy. In response, we have now incorporated the complete list of SSA countries into the search strategy section of the manuscript, using the World Bank regional classification for Sub-Saharan Africa as the authoritative reference. |  |
| 5 | The eligibility criteria should be specified in the main manuscript. Therefore, move the table on eligibility criteria to the main manuscript. | We sincerely thank the editor for highlighting the importance of presenting the eligibility criteria within the main manuscript. We have now moved the full table from the supplementary material into the main text. We appreciate this guidance, as it enhances the clarity and usability of the protocol for readers. |  |
| 6 | In the data analysis sub-section, you need to state that data will be summarised narratively. | We sincerely thank the editor for this helpful suggestion. We have revised the Data Analysis subsection to explicitly state that data will be summarised narratively, in line with the scoping review aim. Tables and figures will be used to present study characteristics and key findings, ensuring clarity and accessibility for readers. |  |
